# Supplementary material for: Reconfigurable SiC gratings in PDMS: a portable approach for atmospheric optical communication networks
Source: Light Sci Appl. 2025 Dec 2;14:393. doi: 10.1038/s41377-025-02060-0 (PMC12669780; doi:10.1038/s41377-025-02060-0)
Supplement: Supplementary file 1 — Supplementary information [file 41377_2025_2060_MOESM1_ESM.docx]

Supplementary information for

**Reconfigurable SiC gratings in PDMS: A portable approach for atmospheric optical communication networks**

Wanzhuo Ma^1, 3 #, *^, Yanwei Fu^1, 3 #^, Dong-Dong Han^2, *^, Keyan Dong^1, 3, *^, Jiaqing Zeng^1, 3^, Qiang Wang^2^, Peng Lin^1^, Yong-Lai Zhang^2, *^, Ye Gu^3^, Zhi Liu^1^, Xianzhu Liu^1^, Huilin Jiang^1^

^1^ Space Optoelectronic Technology Research Institute, Changchun University of Science and Technology, Changchun 130022, China

^2^ State Key Laboratory of Integrated Optoelectronics, Jilin University, Changchun 130012, China

^3^ College of Opto-Electronic Engineering, Changchun University of Science and Technology, Changchun 130022, China

^#^ These authors contributed equally: Wanzhuo Ma, Yanwei Fu.

E-mail: mawz@cust.edu.cn, handongdong@jlu.edu.cn, dongkeyan@cust.edu.cn, [yonglaizhang@jlu.edu.cn](mailto:yonglaizhang@jlu.edu.cn)

**Extended Data**


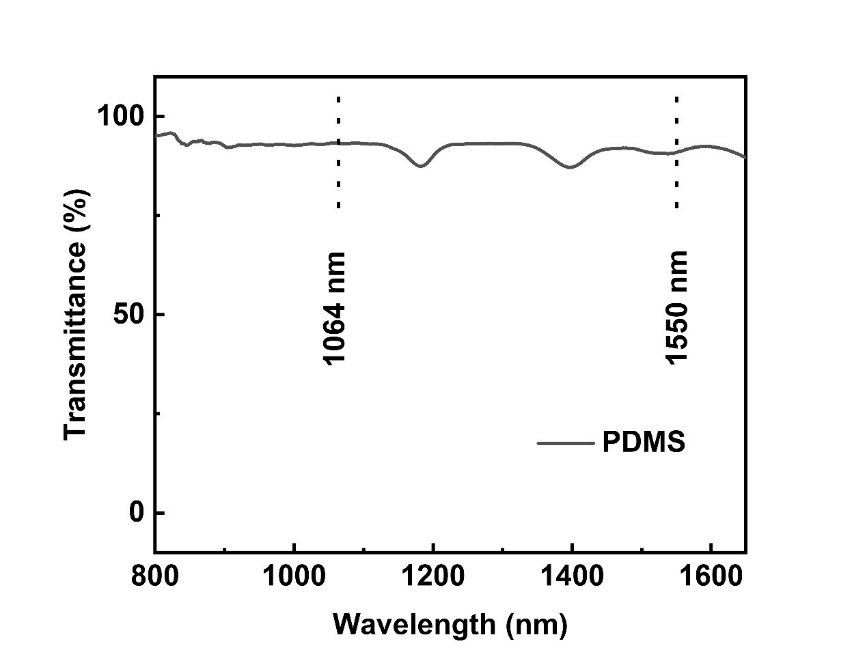


**Fig. S1** Transmittance spectrum of the PDMS film.


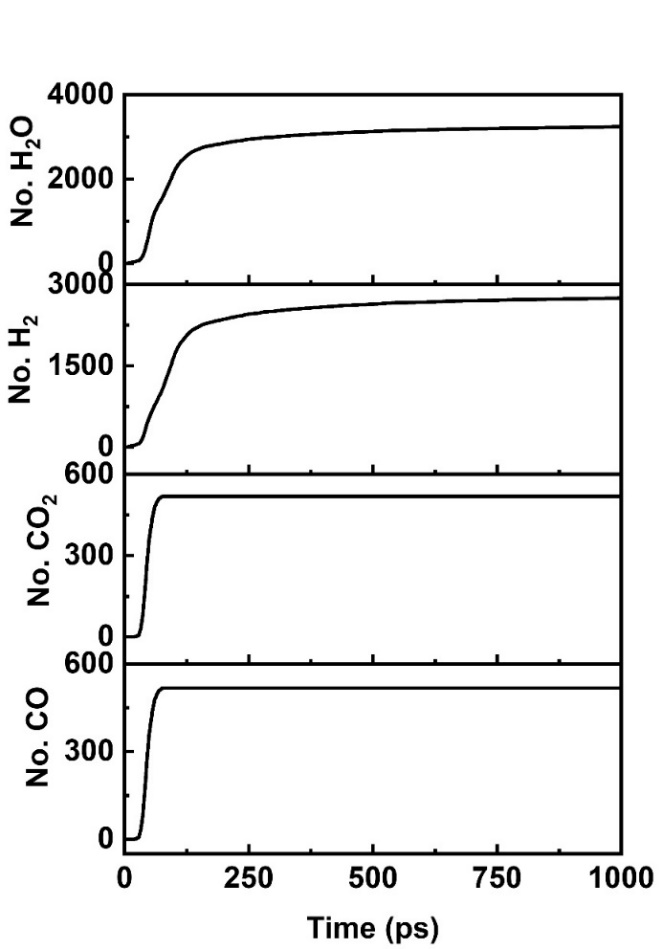


**Fig. S2** Variations in the number of H_2_O, H_2_, CO_2_ and CO molecules in the PDMS system with MD simulation time.


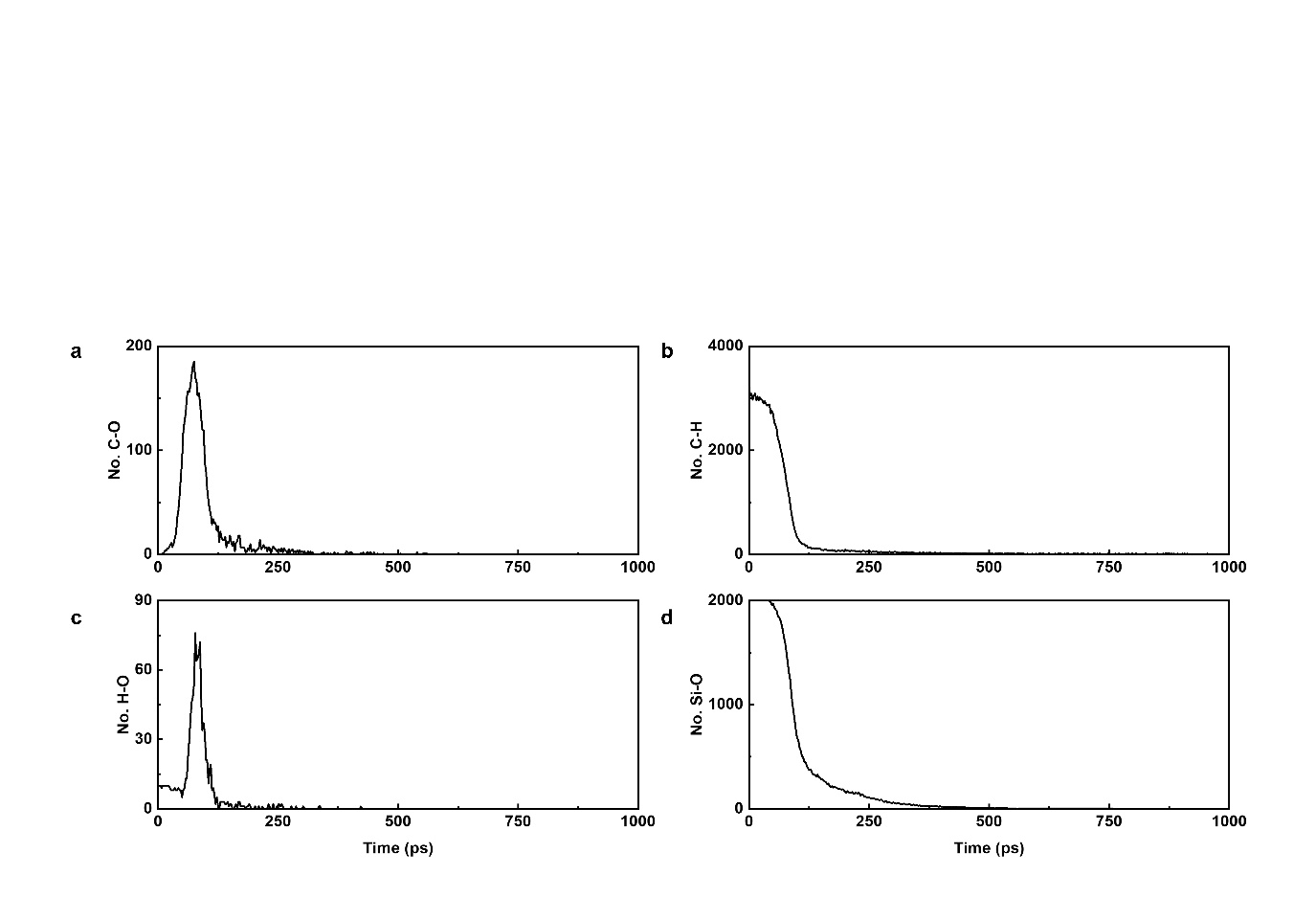


**Fig S3** Variation in the number of (a) C-O, (b) C-H, (c) H-O and (d) Si-O bonds in the PDMS system subjected to thermal-heating with MD simulation time.


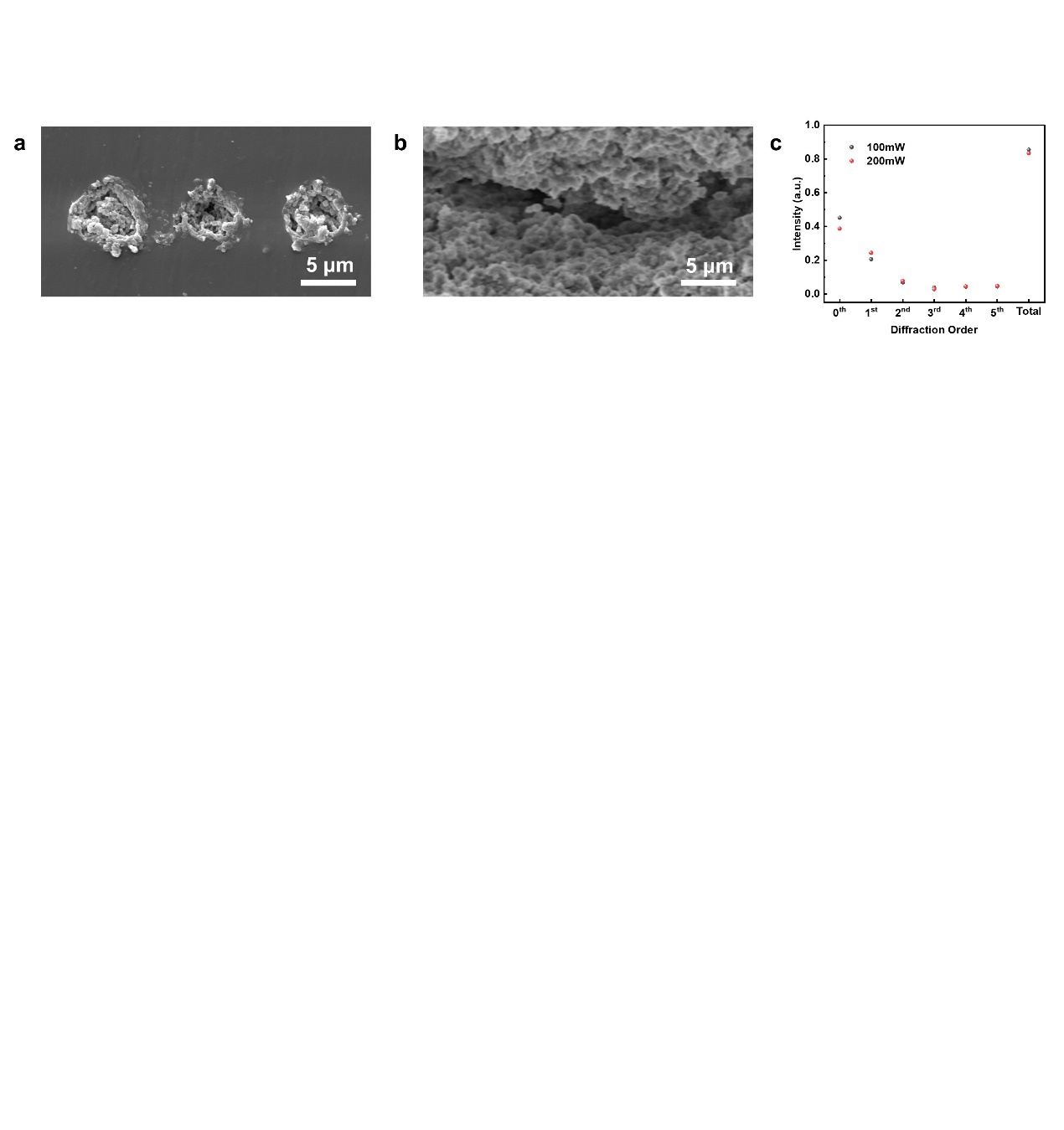


**Fig. S4** Cross-sectional SEM images of SiC gratings fabricated at pulse energies of (a) 1 μJ and (b) 2.5 μJ. The grating thickness increases with energy, while excessive energy leads to feature merging and loss of definition. (c) Intensity distribution of diffracted beams with different laser processing power.


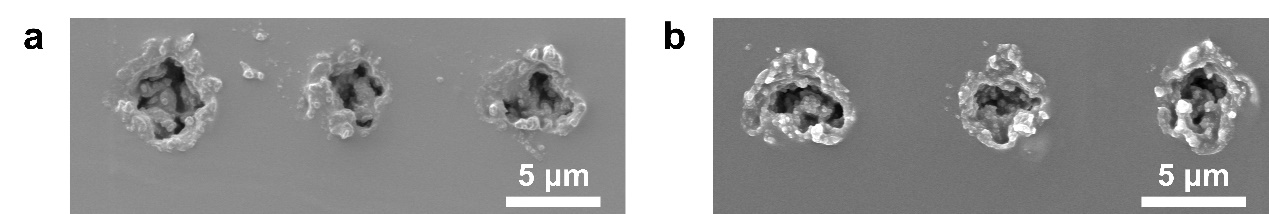


**Fig. S5** Cross-sectional SEM images of SiC gratings fabricated in PDMS with curing ratios of (a) 5:1 and (b) 20:1, showing similar feature sizes under identical laser conditions.


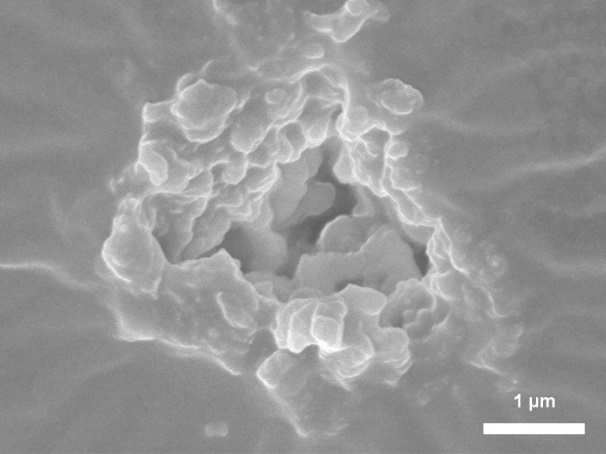


**Fig. S6** High-resolution cross-sectional SEM image of the grating line.


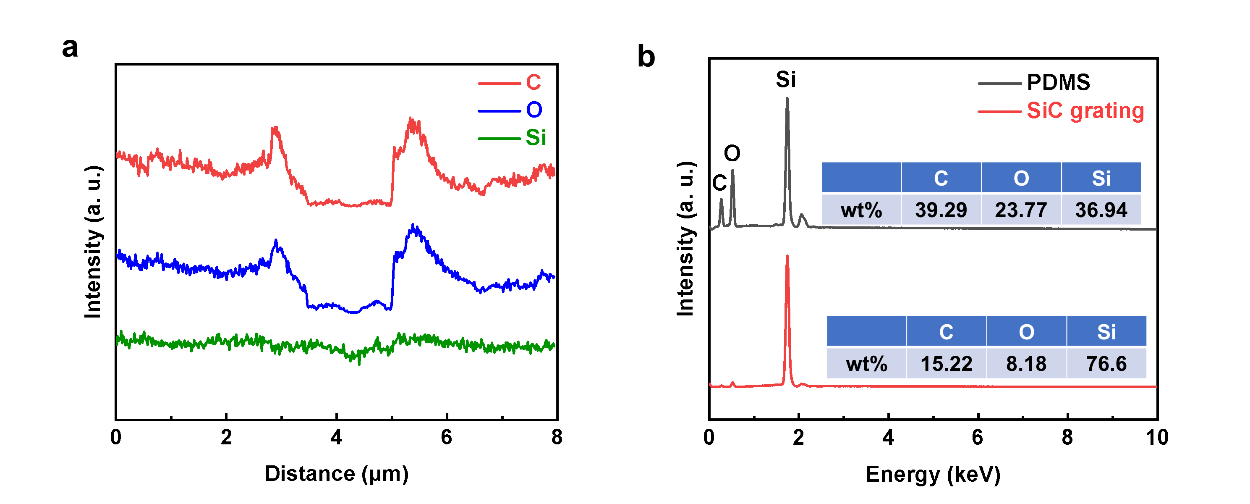


**Fig. S7** (a) EDS line scanning profiles. (b) EDS spectra of the initial PDMS and SiC grating.


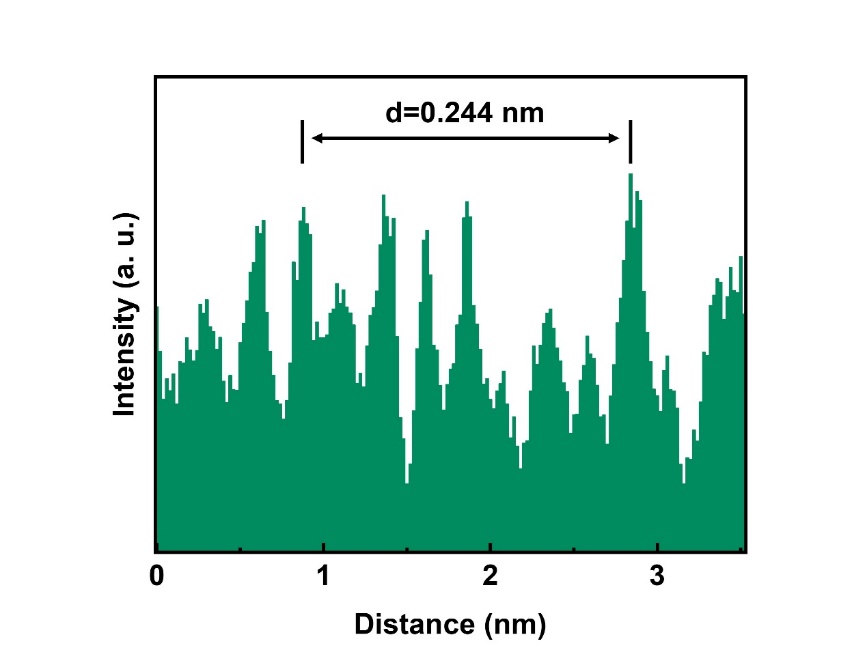


**Fig. S8** Lattice distance calculated from a digital micrograph.


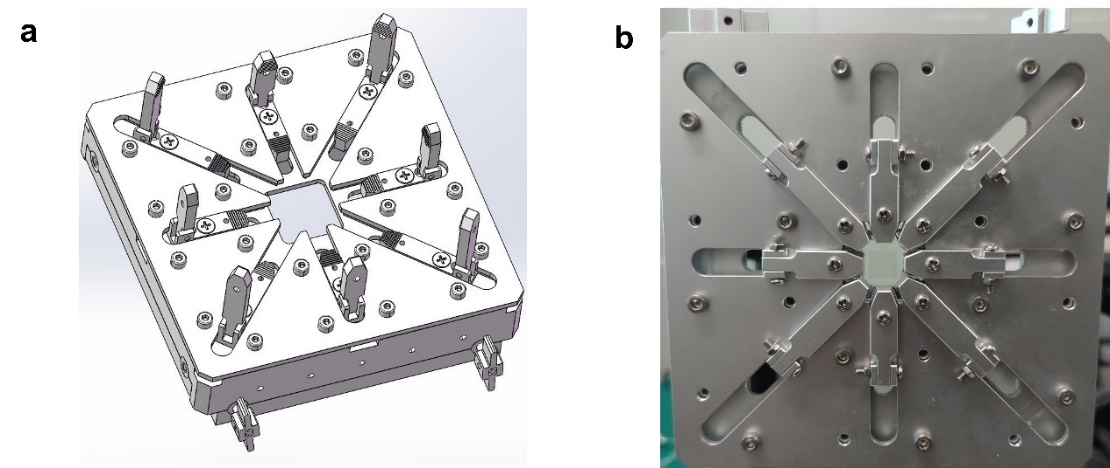


**Fig. S9** (a) Design and (b) photograph of the custom-designed square stretching device.


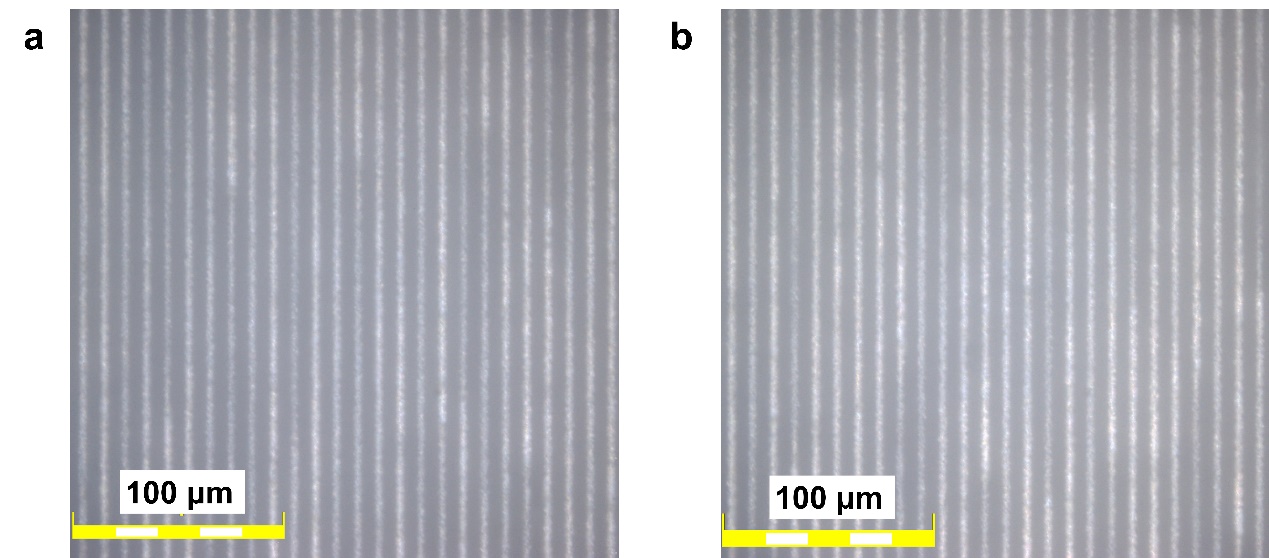


**Fig. S10** CLSM images of embedded SiC gratings (a) before and (b) after 1000 stretching-releasing cycles at 38% strain. No visible cracking or delamination is observed, indicating good mechanical stability of the grating structures during repeated deformation.


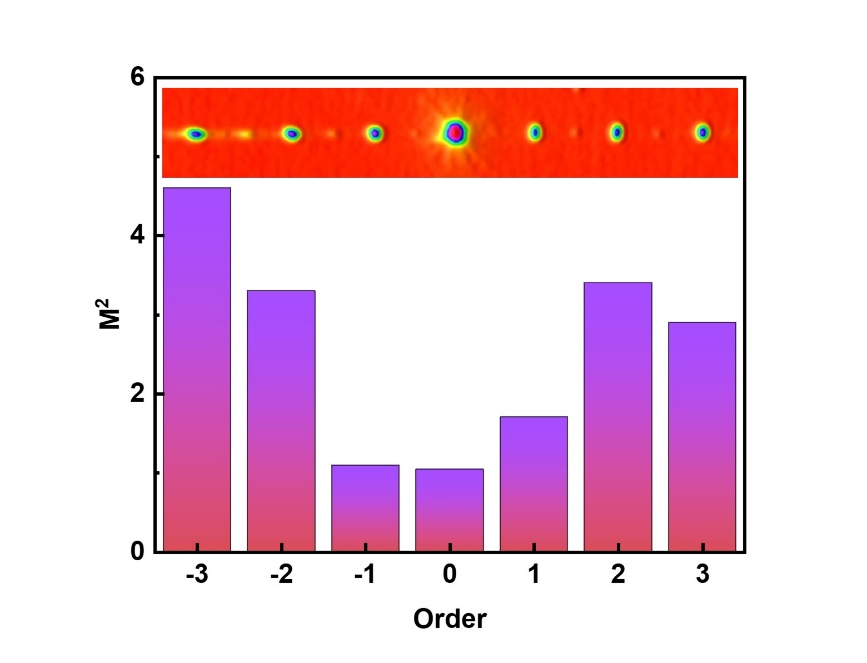


**Fig. S11** Beam quality of the surface 1D diffraction spots.


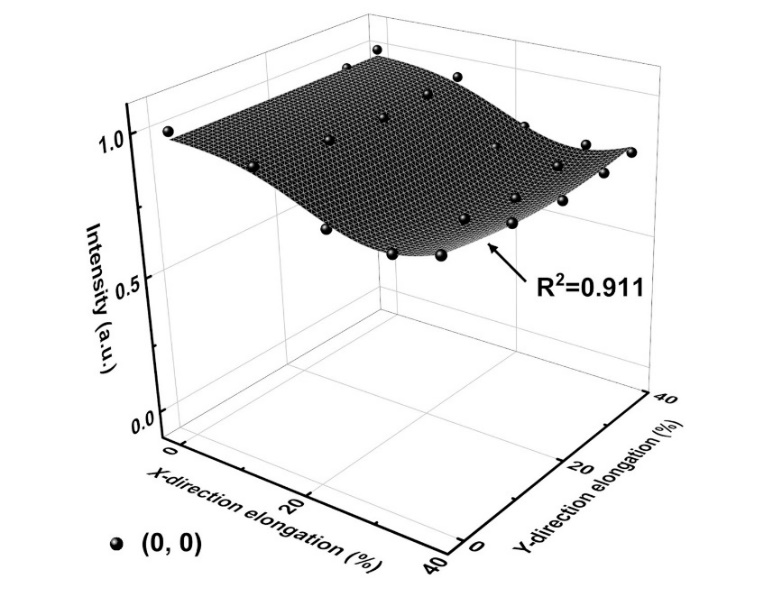


**Fig. S12** Dependence of the (0^th^, 0^th^)-order 2D diffraction efficiency on the mechanical elongation.


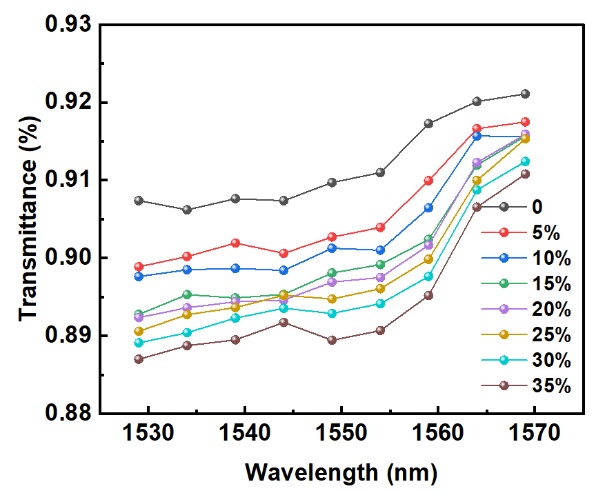


**Fig. S13** SiC grating's transmittance from 1530-1550 nm during dynamic tuning


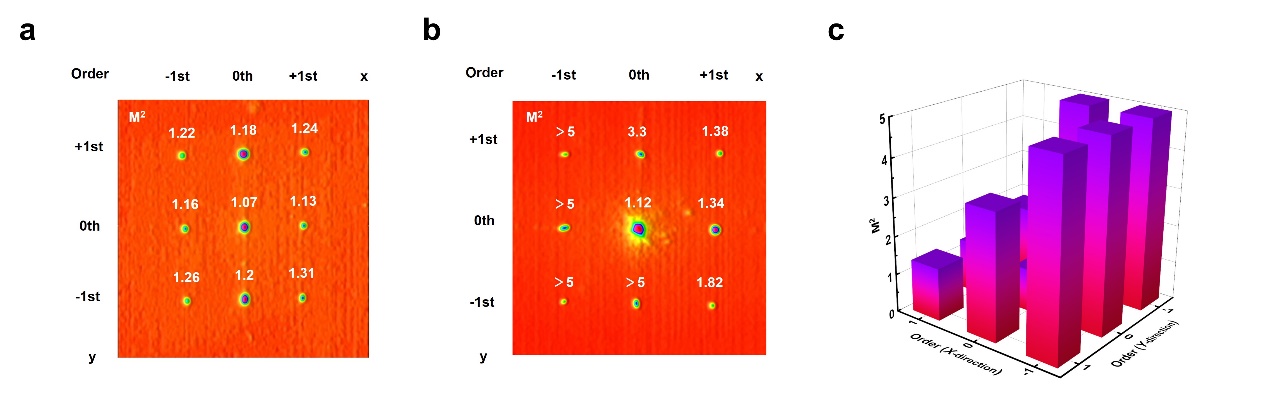


**Fig. S14** (a) Light field distributions of diffraction spots obtained using an internal SiC grating. (b) Light field distributions and (c) 2D beam quality of diffraction spots obtained using surface SiC gratings.


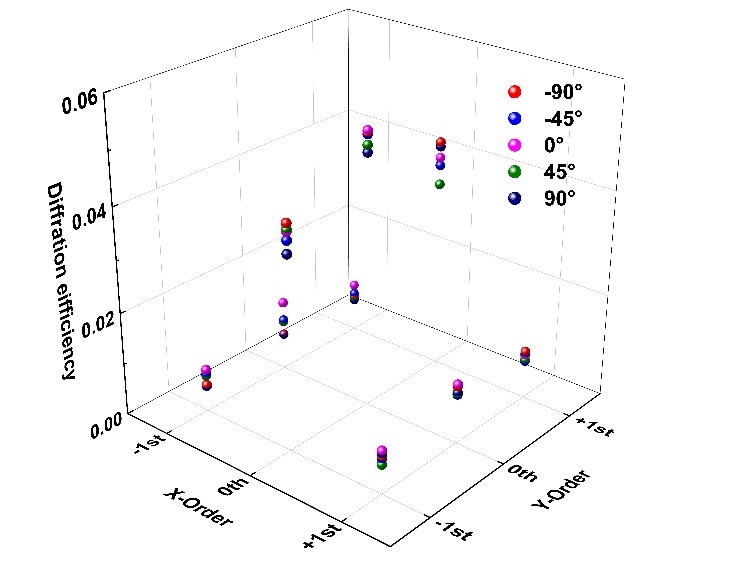


**Fig. S15** Diffraction efficiency of 2D scanning system under different polarization states of laser sources


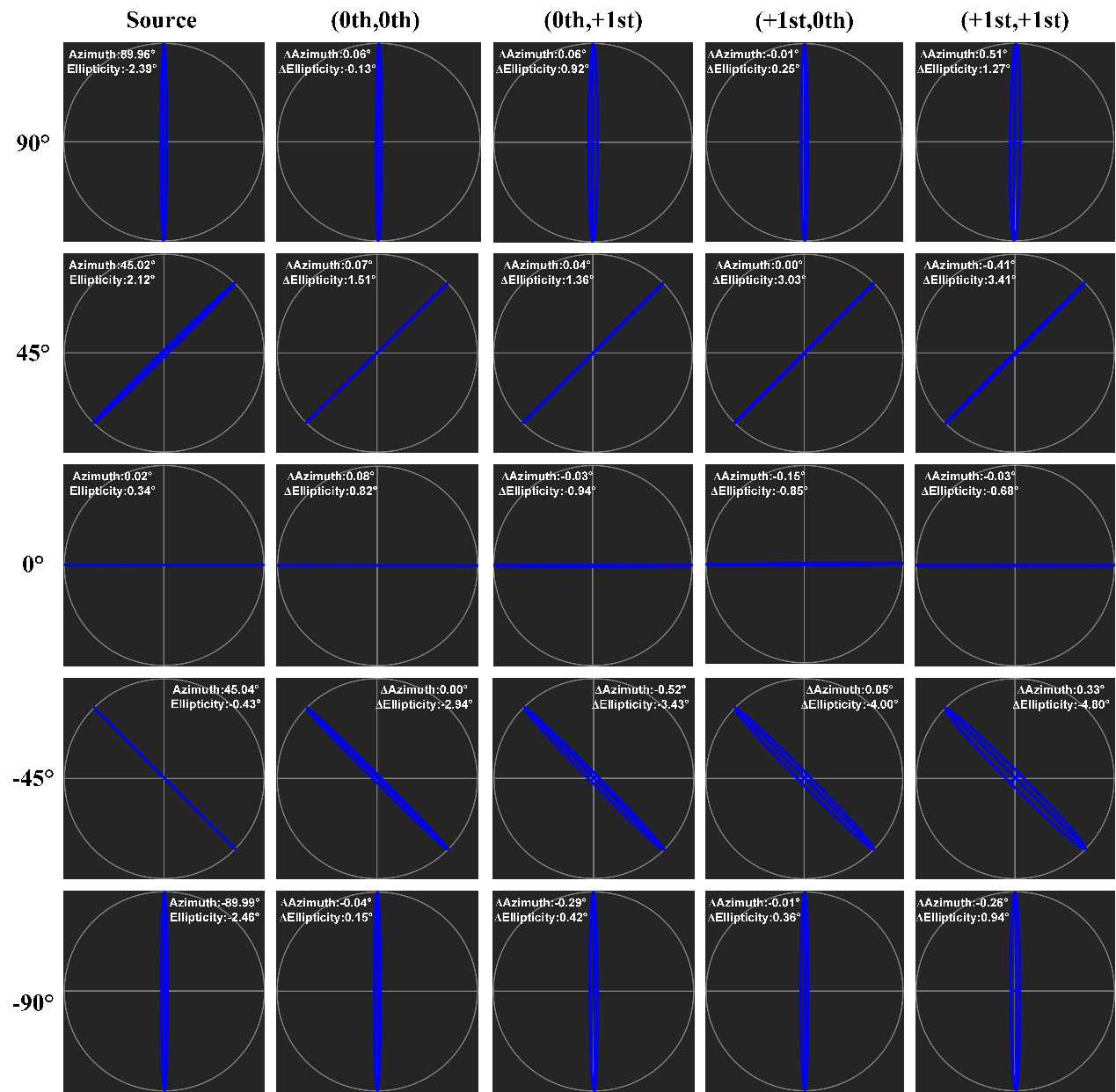


**Fig. S16** Polarization evolution of 2D scanning system under different polarization states of laser sources


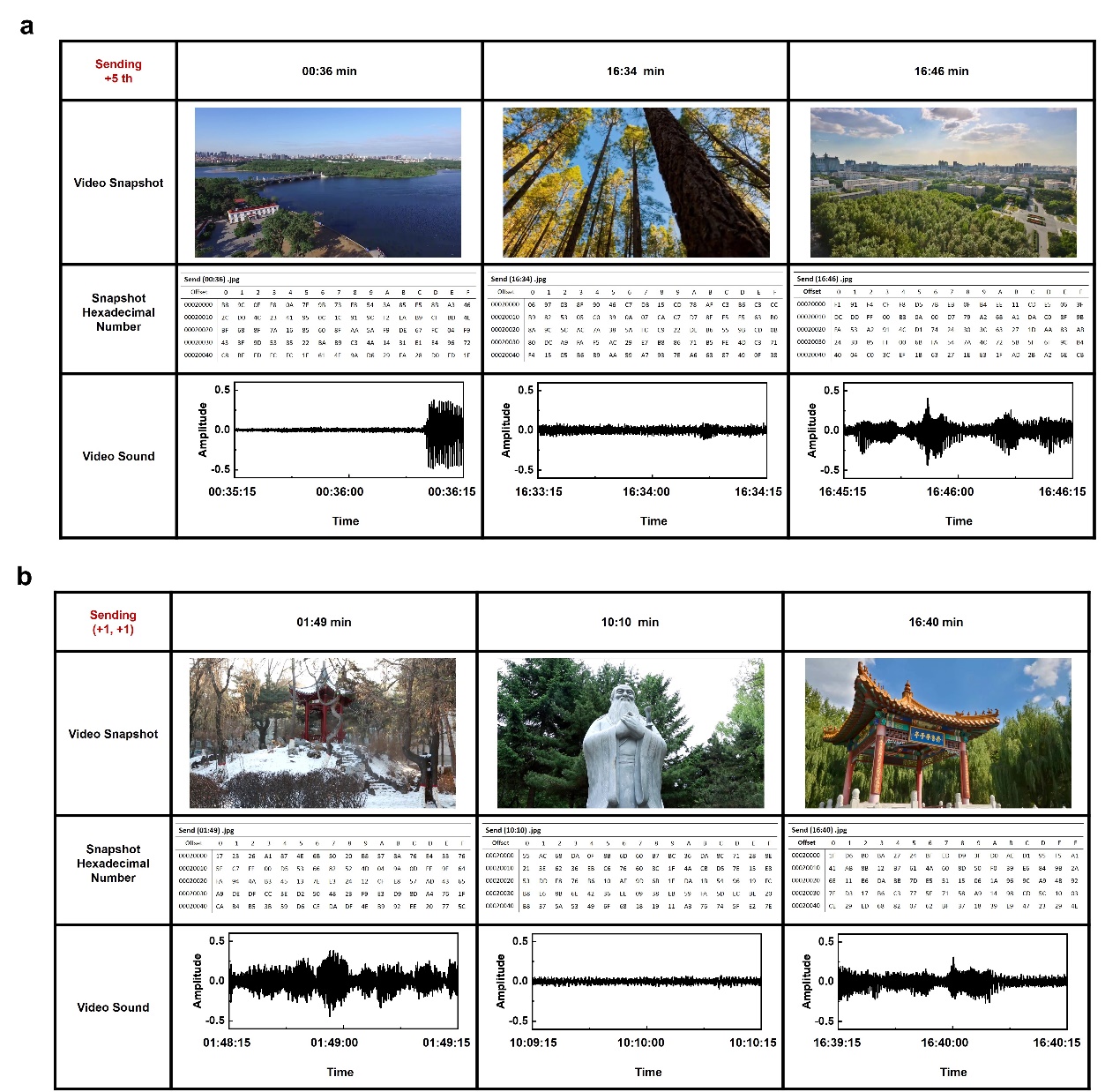


**Fig. S17** Sending data of video snapshots, hexadecimal number snapshots and video sound. (a) Space laser communication system based on a single grating (b) Space laser communication system based on cascaded gratings.


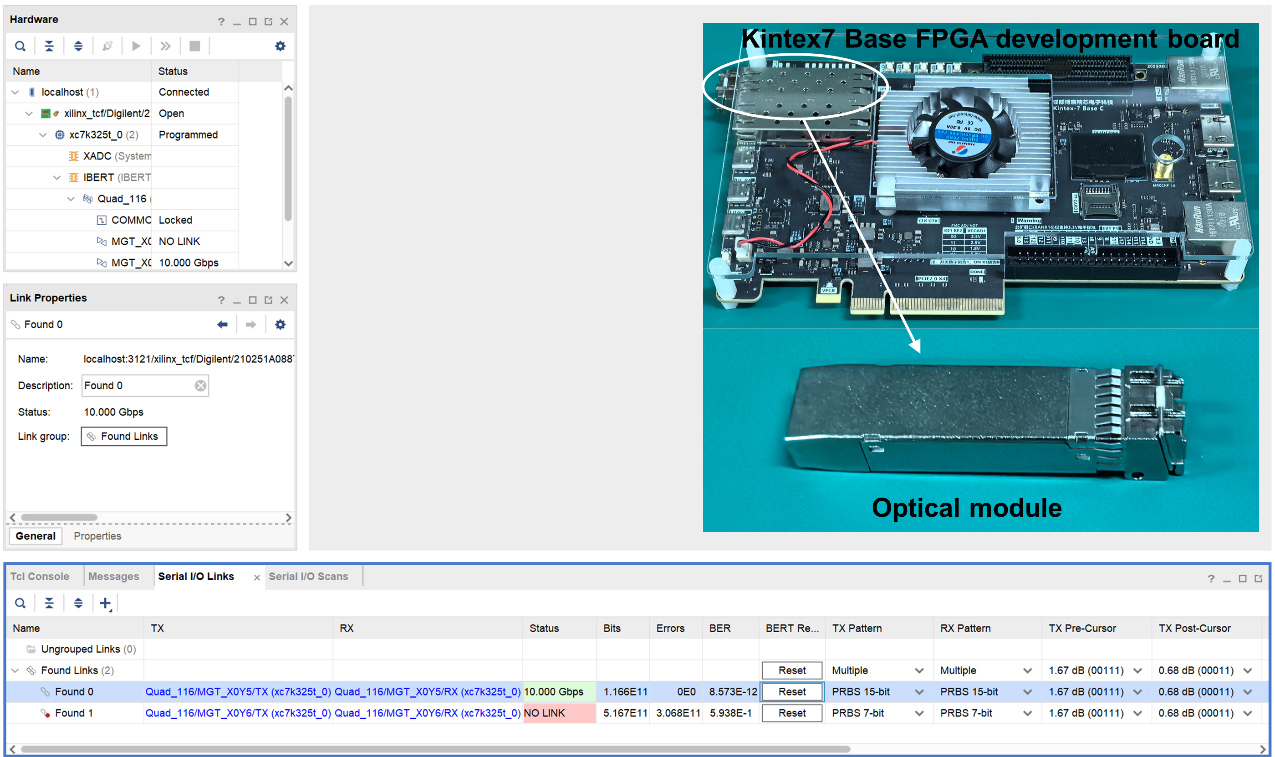


**Fig. S18** Physical object and software interface of the FPGA development board.


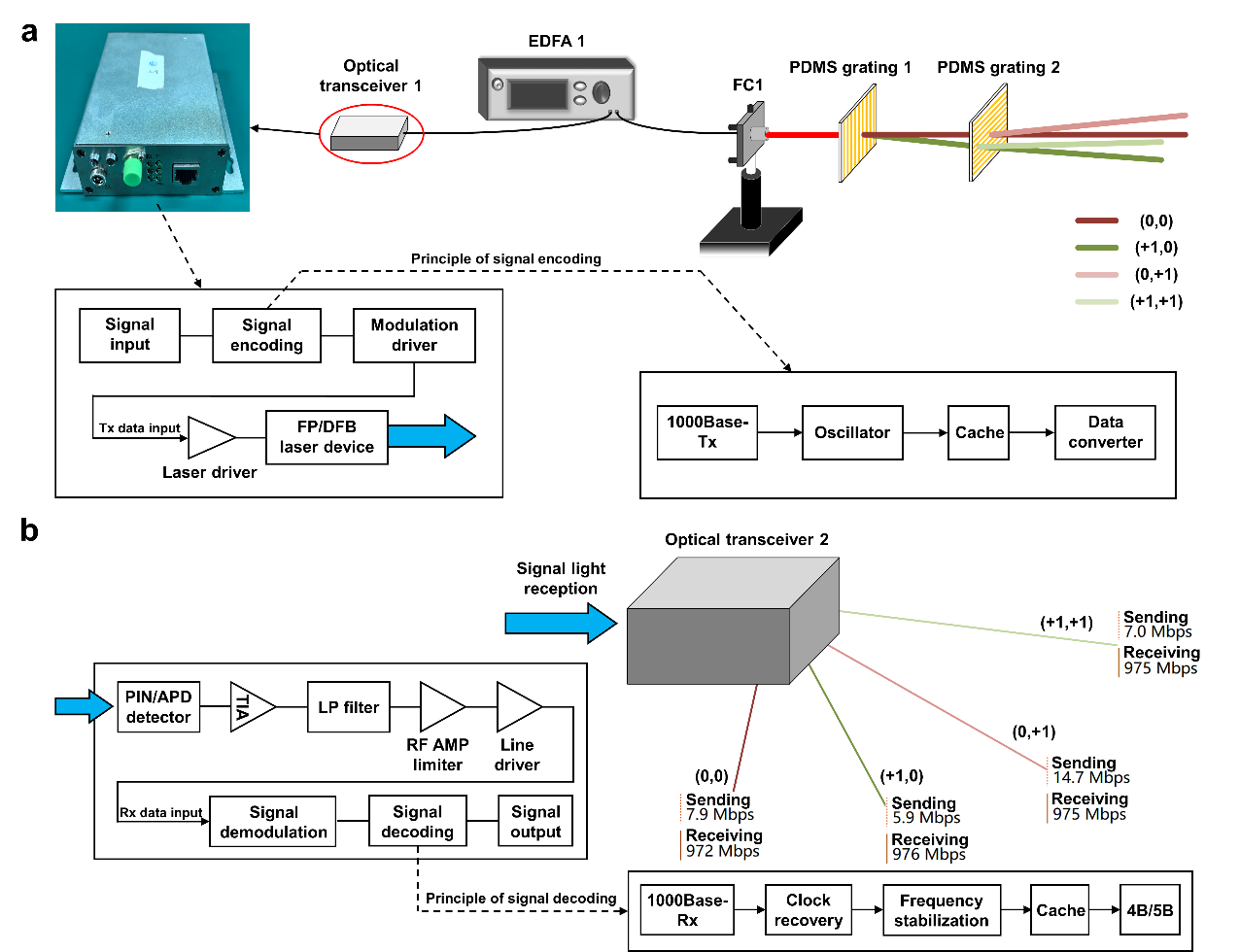


**Fig. S19** Schematic diagram of video transmission using an optical transceiver. (a) Transmitting end. (b) Receiving end.

**
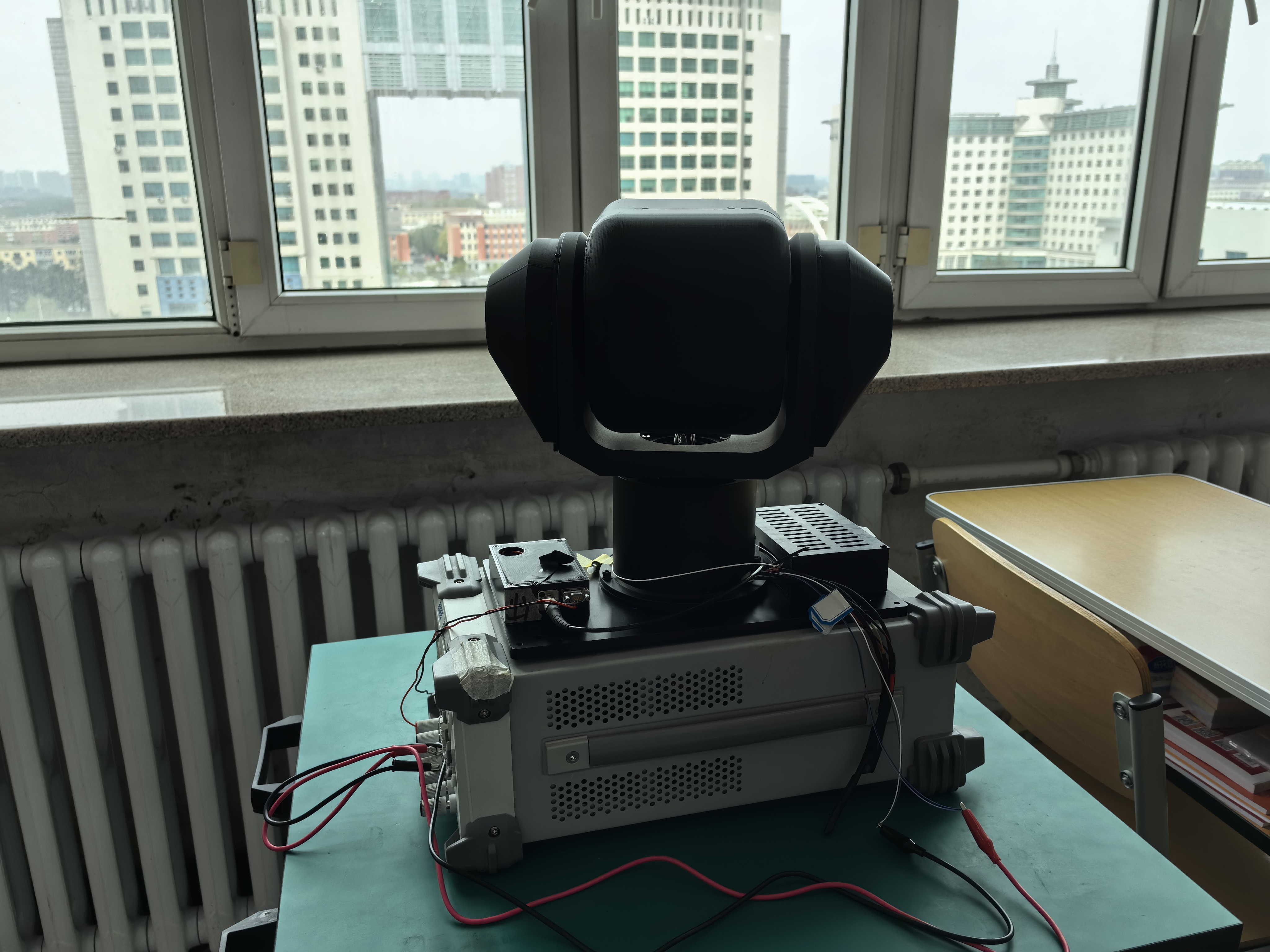
**

**Fig. S20** Home-built FSOC terminal for small platforms.


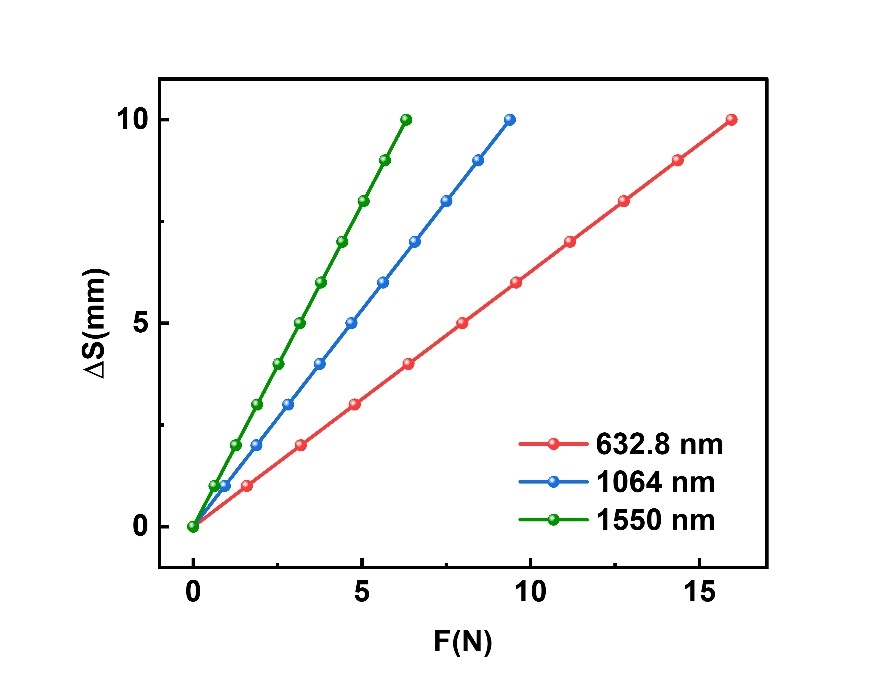


**Fig. S21** Relationship between the variation in the diffraction spot and tensile force.


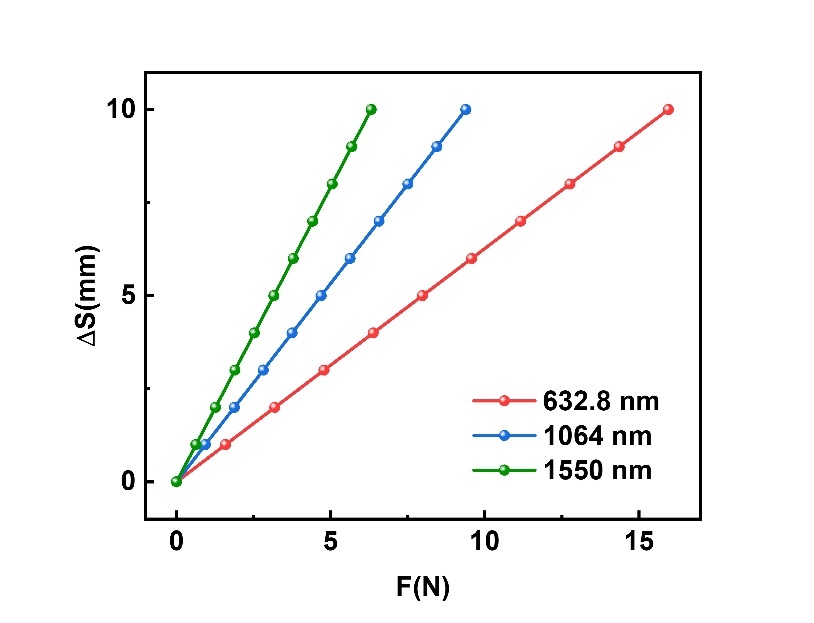


**Fig. S22** Relationship between the variation in the diffraction spot position and stretching force.

**
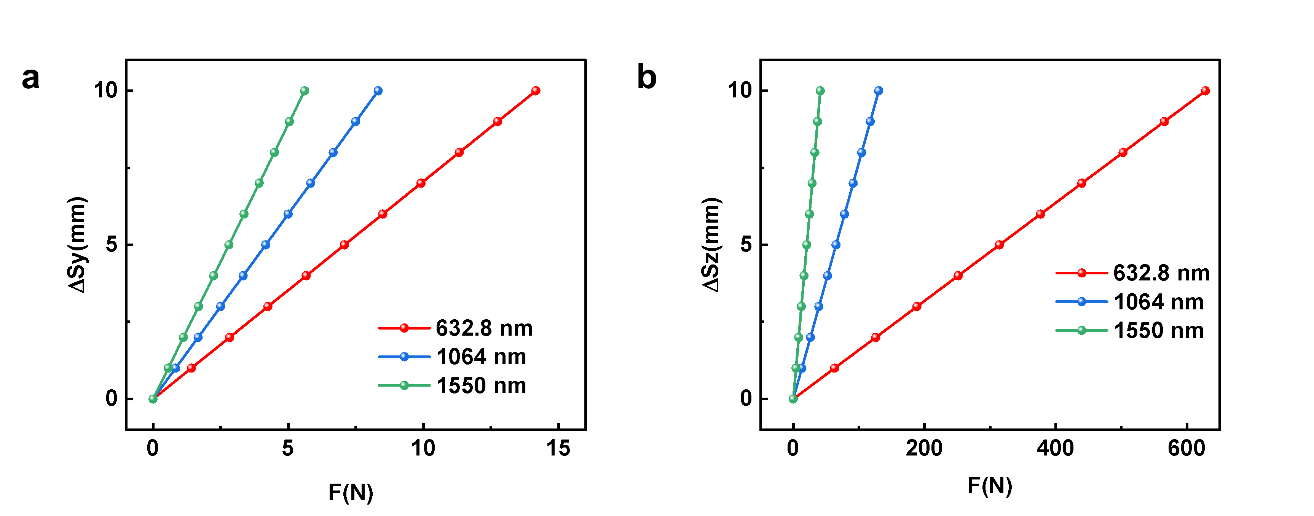
**

**Fig. S23** Relationship between the stretching force and the (a) change in the spot position ΔSy and (b) change in the spot position ΔSz.

**Reconfigurable SiC gratings in PDMS: A portable approach for atmospheric optical communication networks**

Wanzhuo Ma^1, 2 #, *^, Yanwei Fu^1, 2 #^, Dong-Dong Han^3, *^, Keyan Dong^1, 2 *^, Jiaqing Zeng^1，2^, Qiang Wang^3^, Peng Lin^1^, Yong-Lai Zhang^3, *^, Ye Gu^2^, Zhi Liu^1^, Xianzhu Liu^1^, Huilin Jiang^1^

^1^ Space Optoelectronic Technology Research Institute, Changchun University of Science and Technology, Changchun 130022, China

^2^ College of Opto-Electronic Engineering, Changchun University of Science and Technology, Changchun 130022, China

^3^ State Key Laboratory of Integrated Optoelectronics, JLU Region, College of Electronic Science and Engineering, Jilin University, Changchun 130012, China

^#^ These authors contributed equally: Wanzhuo Ma, Yanwei Fu.

E-mail: [mawz@cust.edu.cn](mailto:mawz@cust.edu.cn), handongdong@jlu.edu.cn, dongkeyan@cust.edu.cn, [yonglaizhang@jlu.edu.cn](mailto:yonglaizhang@jlu.edu.cn)

**Abstract**

Free-space optical communication (FSOC) enables high-speed, secure, and scalable data transmission, with great potential for space–ground networks. However, existing FSOC systems predominantly employ point-to-point transmitters, each requiring bulky beam steering devices with complex control mechanisms, which severely limits their feasibility for multi-node micro-platform applications. Here, to address such a challenge, we propose a novel point-to-multipoint FSOC scheme based on reconfigurable SiC gratings, which are directly fabricated in stretchable PDMS films via femtosecond laser-induced carbide precipitation. The reconfigurable SiC transmission gratings are with good transparency (~91.9% at 1550 nm), dynamic beam steering capability (hundred-milliradian level), and an ultralightweight design (single grating: 0.4 g). The SiC fringes are specially fabricated within the internally symmetric region of the PDMS film to mitigate the structure distortion during stress regulation, significantly enhancing the long-range transmission capability in degraded atmospheric channels. The system supports 1-to-7 and 1-to-9 dynamic optical communication for 1D and 2D configurations, respectively. In a state-of-the-art 225-meter outdoor experiment, the system achieves reliable 10 Gbps transmission for each node. This portable FSOC system overcomes the limitations of conventional systems, enabling scalable and flexible multibeam steering. This approach establishes a robust foundation for long-range, multinode and high-capacity FSOC networks among spatial micro-platforms such as unmanned aerial vehicles and micro-satellites.

**Introduction**

Free-space optical communication (FSOC) is a revolutionary technology for wireless data transmission via optical signals, with advantages such as high bandwidth, great versatility, and high security ^1-3^. In particular, FSOC is highly suitable for backbone networks, access networks, and subnets within space–ground integrated information systems ^4,5^. Conventionally, as FSOC systems work in point-to-point mode, for a space network with N nodes, each node requires at least N-1 optical transmitters to achieve real-time communication between nodes. This point-to-point mode relies on coarse beam steering, which is achieved through the use of mechanical turntables and fine adjustments with steering mirrors ^6-8^, resulting in a high system weight (*e.g.*, several kilograms to tens of kilograms). Although great efforts have been devoted to miniaturizing these systems using microelectromechanical systems or liquid crystal devices, bulky control systems are still needed ^9,10^. Establishing FSOC networks on low-load platforms, such as microsatellites and unmanned aerial vehicles, is important for various applications but is associated with significant challenges to ensure real-time, stable, and scalable data exchange. Moreover, as the number of transceivers and the complexity of conventional FSOC networks increase, efficient multipoint communication among miniature devices becomes increasingly critical.

To overcome these limitations, researchers have proposed advanced point-to-multipoint FSOC technologies, in which the point-to-point mode is replaced by single-input and multiple-output laser communication components with beam-splitting capabilities ^11,12^. Several methods, such as the use of double prisms and optical phased arrays, have shown promise in achieving large steering angles and high optical gains ^11,12^, greatly reducing the number of transceivers needed. However, these approaches still rely on bulky phase control or beam deflection systems. Thus, there is an urgent need for compact, portable point-to-multipoint FSOC technologies to facilitate laser networking on small-scale platforms.

Recently, passive microstructure optoelectronic devices represented by metasurfaces have shown great potential for miniaturization and integration, making them promising for both point-to-point and point-to-multipoint FSOC applications ^13-15^. They represent the current state-of-the-art for manipulating the amplitude, phase, and polarization of light, enabling high-speed Gbps data transmission ^16-20^. However, metasurfaces have low efficiency and stringently depend on the precision of the fabrication process, greatly influencing the beam quality. Thus, metasurface-based beam steering devices are currently unable to support long-distance laser transmission through atmospheric channels, and are currently limited to short-range communication scenarios within indoor or laboratory environments ^21,22^. In contrast, gratings have advantages such as high efficiency, substantial integration capacity, and minimal beam distortion and are thus more suitable for long-distance transmission, enabling cutting-edge applications in inline optical delay lines, user localization, and multiplexed transmission ^23-26^. Nevertheless, traditional grating-based systems are constrained to static targets owing to their reliance on spatial–wavelength mapping. To address these limitations, reconfigurable gratings have been developed with the help of advanced manufacturing techniques such as e-beam writing, ion-beam lithography, and polymer-induced phase separation. Due to the flexible substrate, the grating period can be reconfigured in real-time under external driving forces, which facilitate controlled multibeam steering ^27-31^. These innovations with tunable gratings have extended their applications in fields such as soft robotics, passive isothermal films, flexible sensors, and line-scanning microscopy ^32-35^. However, practical issues with reconfigurable gratings, such as uneven substrate deformation during operation, usually result in beam distortion at higher diffraction orders, which makes such systems ineffective for laser transmission in turbulent atmospheric channels. To achieve precise and distortion-free multibeam transmission in practical point-to-multipoint FSOC systems, the development of reconfigurable gratings through innovative designs and advanced manufacturing techniques is highly desirable.

In this work, reconfigurable SiC gratings within stretchable PDMS films were developed via femtosecond laser-induced carbide precipitation for high-capacity point-to-multipoint FSOC systems. The grating structure ensures excellent beam quality during square stretching, enabling low-distortion transmission of high-order diffracted beams. Additionally, dynamic steering for point-to-multipoint FSOC systems was achieved using 1D and cascaded 2D diffraction grating systems. Subsequently, validation in a simulated atmospheric channel confirms the turbulence resistance of the proposed gratings for 0^th^-order and higher-order beams, and in outdoor experiments, 1D (1-to-7) and 2D (1-to-9, 3×3) high-speed links were established over a 225-meter channel under real atmospheric conditions. The developed approach offers marked advantages in terms of the number of nodes, scalability, and transmission distance, and represents a transformative technique for advancing portable beam-steering devices in FSOC applications.

**Results**

**Design principles.** The aim of the proposed design is to create a portable and high-capacity point-to-multipoint FSOC system with properties such as long-distance transmission capability, which cannot be achieved with metasurfaces, and portability, which is a limitation of conventional servo-driven fast-steering mirrors. This design facilitates the establishment of FSOC networks across compact ground and aerial platforms, such as vehicles, buildings, unmanned aerial vehicles, and airships (Fig. 1a_1_). A core aspect of the design is the use of reconfigurable gratings that dynamically split single-channel laser beams into multiple laser channels (Fig. 1a_2–3_). Each channel carries high-speed information, enabling simultaneous communication with multiple nodes. For example, a 1D point-to-multipoint linear array system (link 1) can be used to create a 1-to-3 FSOC link with one transmitting point (e.g., a tower) and three receiving points (e.g., a building, a car and an airship). Furthermore, this concept can be extended to 2D point-to-multipoint FSOC by cascading two 1D systems (link 2). In this configuration, transmission diffraction gratings are used to enable laser steering and communication with a planar array, significantly increasing the node capacity and steering coverage. For example, a 1-to-9 planar array FSOC link with one transmitting point (e.g., an airship) and nine dynamic receiving points (e.g., unmanned aerial vehicles) can be established. The period of the transmission grating and the deflection angle of the transmitted laser beam are rapidly tunable through reversible mechanical stretching. Owing to this mechanical tunability, the system can adapt to dynamic communication scenarios. Additionally, the cascaded configuration can seamlessly transition from linear to planar steering and communication, employing the system for diverse and complex network configurations.

One of the critical challenges in this design is to ensure the stability and uniformity of tunable gratings during mechanical manipulation, since instability directly impacts the performance of point-to-multipoint FSOC systems. Finite element analysis was used to obtain the information presented in Fig. 1b. Conventional flexible gratings created through surface processing often suffer structural failure during stretching or recovery due to the mismatch in the elastic moduli between the fringes and unmodified regions (Fig. 1b_1_). This failure results in gross distortion of the surface transmission grating, which degrades the quality of the diffracted laser beam and reduces the effective transmission distance of the FSOC link. To overcome these limitations, internally processed gratings that retain a consistent planar structure during reversible stretching are incorporated into the proposed design (Fig. 1b_2–3_). This approach effectively prevents beam distortion caused by uneven substrate stretching. Furthermore, a square stretching method is employed for the grating instead of the conventional bidirectional stretching approach (Fig. 1b_3_). With this innovative stretching method, the internal grating can be simultaneously regulated across all directions, which effectively addresses edge shrinkage and fringe distortion. As a result, the structural integrity of the grating is preserved, and the high beam quality is ensured. By addressing structural stability and beam distortion issues, this reconfigurable grating design offers a scalable, high-performance solution for point-to-multipoint FSOC systems. This design establishes a robust foundation for the compact, lightweight, and dynamic beam-steering devices suitable for diverse communication platforms.


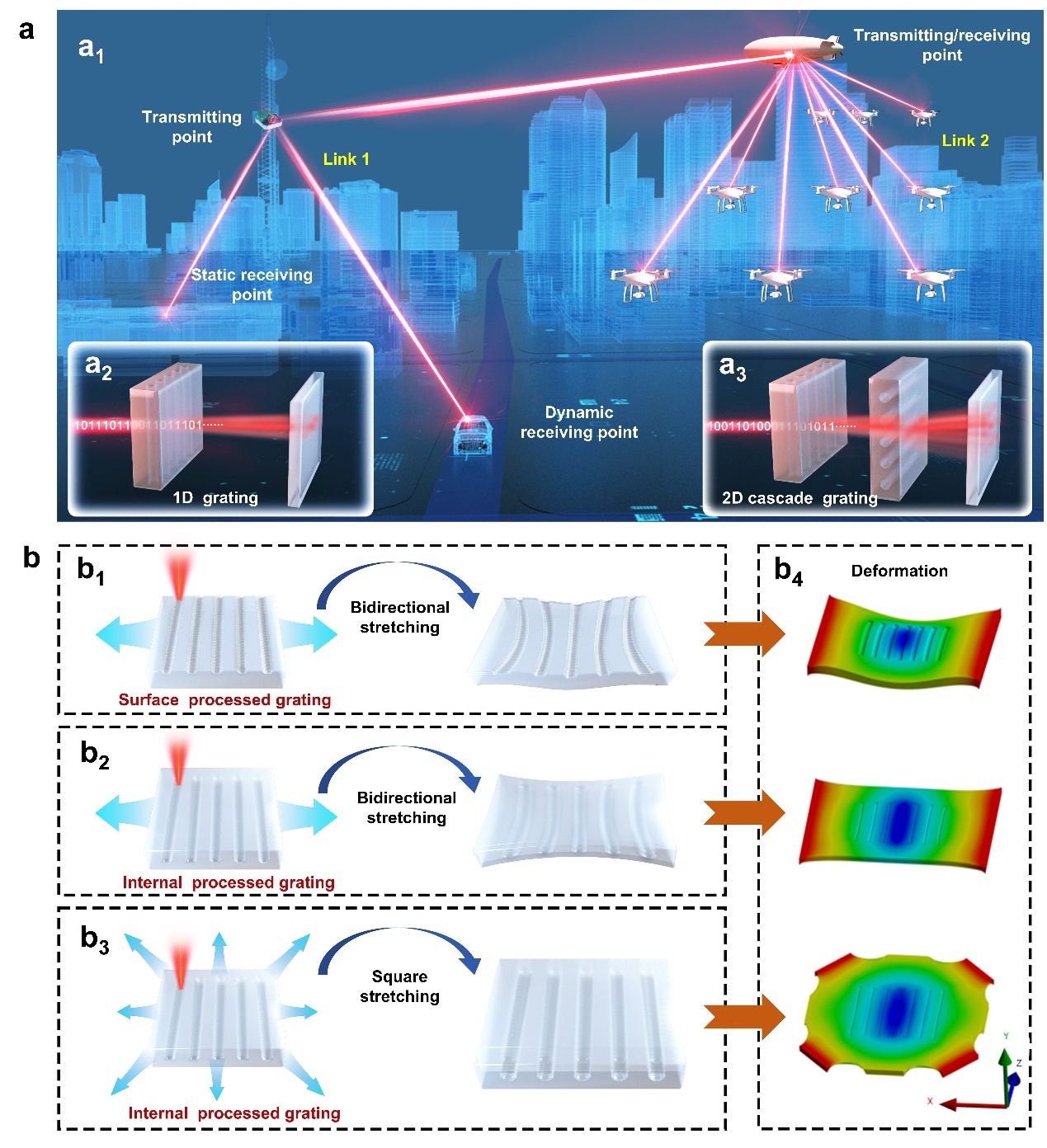


**Fig. 1. Schematics of the point-to-multipoint FSOC utilizing reconfigurable internal gratings. a** Conceptual illustration. **b** Schematic diagrams and finite element analysis illustrating the mechanical manipulation mechanisms for surface and internal gratings.

**Fabrication of reconfigurable SiC gratings.** The reconfigurable grating serves as the core device in this work, enabling multi-beam steering and one-to-multiple communication. SiC was selected for fabricating the reconfigurable gratings because of its high transmittance at 1550 nm, which is a critical wavelength for efficient laser steering and communication in FSOC systems. In addition to its excellent optical properties, SiC exhibits superior thermal stability, mechanical strength, and chemical resistance, making it an ideal candidate for developing robust and reliable gratings ^36-40^. However, the intrinsic rigidity and brittleness of SiC pose great challenges for applications involving stretching. The mismatch between the high stiffness of SiC and the elasticity of stretchable substrates often leads to cracking or delamination under mechanical stress. Consequently, the integration of SiC into a stretchable matrix requires the use of innovative techniques to retain its optical properties while ensuring the mechanical tunability of the system. Addressing these challenges is vital for developing SiC-based gratings that balance the rigidity for optical performance and the flexibility for dynamic applications. A stretchable internal SiC grating is fabricated within a PDMS film using femtosecond laser direct writing (Fig. 2a). The fabrication process begins by focusing the femtosecond laser inside the PDMS film, where the energy density reaches 10^12^ W·cm^-2^. The transmittance of the PDMS film at 1064 nm is approximately 91% (Fig. S1), indicating that the interaction between the femtosecond laser and the PDMS is governed by a multiphoton absorption process. This absorption process rapidly increases the temperature in the laser-irradiated zone, initiating pyrolysis to convert PDMS into SiC. Furthermore, the programmable nature of femtosecond laser processing enables the precise patterning of SiC gratings within the PDMS film while leaving untreated regions transparent.

In this work, classic molecular dynamics (MD) simulations were performed to investigate the structural transformation of a PDMS polymer subjected to laser heating at the molecular level (Figs. 2b-c). Fig. S2 shows the numbers of H_2_O, H_2_, CO_2_ and CO molecules formed in the PDMS system subjected to thermal heating in MD simulations. On the basis of the curves, several stages can be roughly identified. The first stage is described by the initial part of the curves, in which there is no significant change in the number of small molecules. In this stage, the PDMS polymer is not dissociated. The second stage is characterized by a steep increase in the number of small molecules. In this stage, the PDMS polymer is dissociated, and small molecules form within the PDMS. At this point, the number of molecules can be sorted as follows: H_2_O > H_2_ > CO_2_ > CO. The last stage is characterized by an approximately constant number of small molecules, indicating the termination of the formation of these molecules. Fig. 2c and Fig. S3 show the variations in the numbers of Si‒C, C‒O, C‒H, H‒O and Si‒O bonds in the PDMS system subjected to thermal heating. The numbers of Si-C, C-H and Si-O bonds in the PDMS system decrease in the early stage, indicating the rapid dissociation of the PDMS polymer. In contrast, there are steep increases in the numbers of C-O and H-O bonds in the system, indicating the formation of small CO, CO_2_ and H_2_O molecules. Following this stage, the numbers of C-O and H-O bonds suddenly decrease due to the removal of the as-formed small CO, CO_2_ and H_2_O molecules. The number of Si‒C bonds increases, then remains constant, and then increases again, which indicates the nucleation of Si‒C crystals.

The laser-treated region in an internal SiC grating embedded within a PDMS film appears white‒gray in color, in contrast to the transparent PDMS (Fig. 2d_1_). The SiC grating is lightweight and compact, measuring approximately 1 cm × 1 cm × 0.08 cm and weighing approximately 0.4 g, making it ideal for applications in small unmanned aerial vehicles and satellites. Additionally, the internal SiC grating demonstrates remarkable flexibility (Fig. 2d_2_). When the internal SiC grating is illuminated with white light, vivid colors are observed within its structure due to chromatic dispersion (Fig. 2d_3_). The internal SiC grating also exhibits a high transmittance of ~91.9% at 1550 nm (Fig. 2e), which is critical for ensuring laser steering and good communication quality in FSOC systems.

Furthermore, the structural and compositional changes induced by femtosecond laser processing were thoroughly characterized. The confocal laser scanning microscopy (CLSM) image reveals that the grating period is approximately 10 μm; the untreated PDMS appears white, and the laser-treated SiC regions appear gray (Fig. 2f). A cross-sectional scanning electron microscopy (SEM) image of the grating in the PDMS film was also captured to investigate the effects of femtosecond laser pyrolysis (Fig. 2g). The untreated PDMS exhibited a flat surface morphology, whereas the laser treatment induced voids within the PDMS film. The laser-induced voids have an average width of ~3.3 μm and are spaced ~6.2 μm apart, defining the effective thickness and periodicity of the embedded SiC grating. This thickness increases with laser pulse energy—from ~3.3 μm at 0.5 μJ to ~5.6 μm at 1 μJ—while excessive energy leads to merging of adjacent features and loss of grating definition (Fig. S4). The voids exhibit nanostructured morphologies, which contribute to the optical modulation properties of the grating. To evaluate the influence of PDMS curing ratio on laser writing performance, we tested formulations of 20:1, 10:1, and 5:1. Cross-sectional SEM images showed similar feature sizes across all cases, suggesting minimal impact of curing ratio under the selected processing conditions (Fig. S5). High-resolution cross-sectional SEM images confirm the absence of ledge roughness or edge defects along the grating lines, indicating excellent structural uniformity (Fig. 2h and Fig. S6). Moreover, energy-dispersive X-ray spectroscopy (EDS) analysis was conducted to analyze the compositional changes in the PDMS induced by femtosecond laser direct writing. The EDS distribution map and line scan of the laser-treated region are presented in Fig. 2h and Fig. S7. The untreated PDMS contains approximately 39.29% carbon, 23.77% oxygen, and 36.94% silicon, whereas the laser-treated region contains 15.22% carbon, 8.18% oxygen, and 76.6% silicon. The considerable reduction in the carbon and oxygen contents suggests the breaking of C–O and C–Si single bonds during laser processing, which facilitates the formation of SiC. Furthermore, high-resolution transmission electron microscopy (HRTEM) analysis confirms the formation of SiC in the laser-treated zone, with a lattice fringe spacing of 0.244 nm, corresponding to the SiC crystalline plane (Fig. 2i and Fig. S8). These results demonstrate that femtosecond laser pyrolysis effectively converts PDMS into high-quality SiC, enabling the fabrication of stretchable internal gratings with excellent optical properties.

**
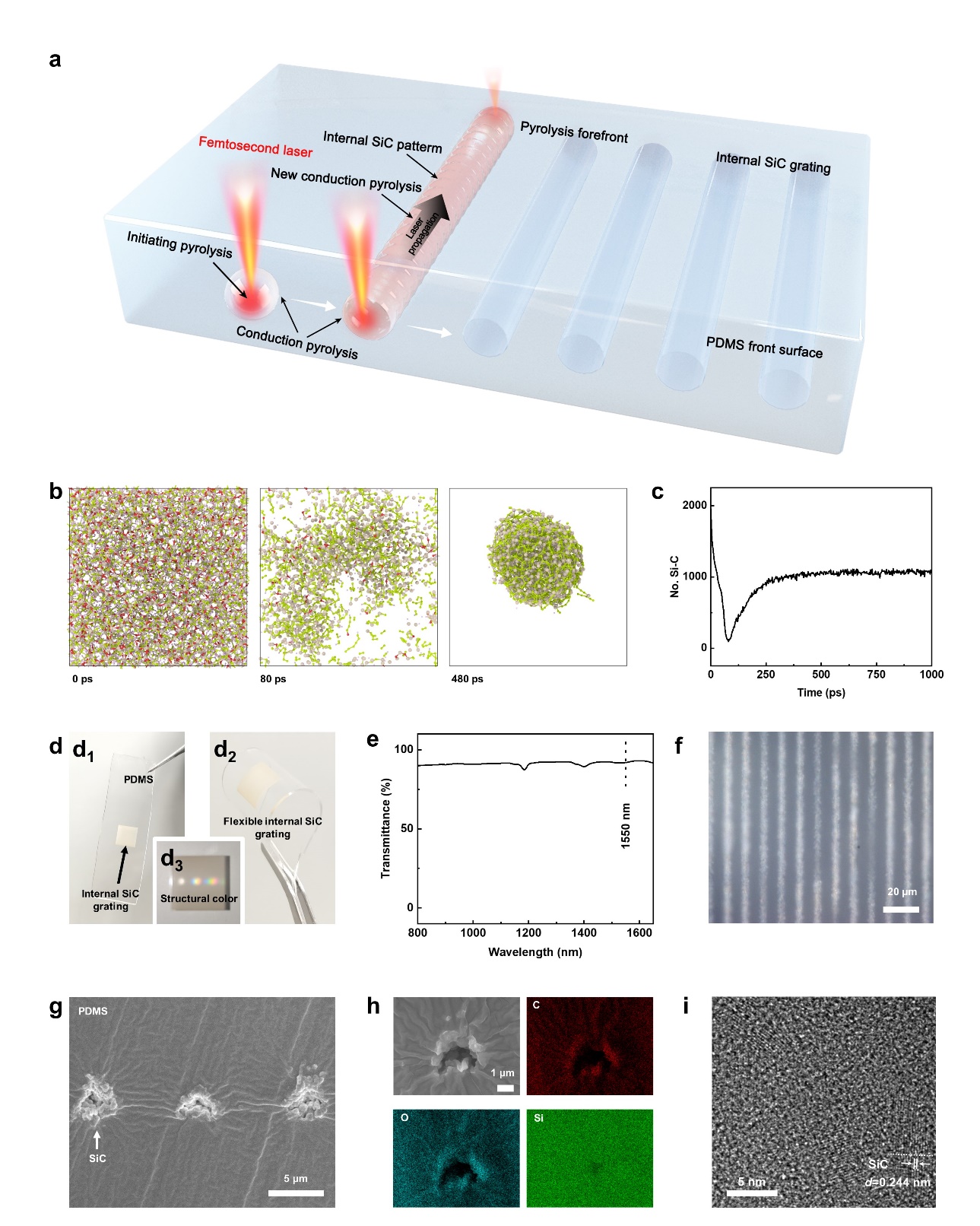
**

**Fig. 2. Fabrication and characterization of stretchable** **internal SiC gratings. a** Schematic representation of the fabrication process for stretchable internal SiC transmission gratings. **b** Formation mechanism of SiC. The atoms are shown as stick models (gray: silicon; green: carbon; red: oxygen; pink: hydrogen). **c** Variation in the number of Si‒C bonds in the PDMS system subjected to thermal heating with increasing MD simulation time. **d** Photographs. **e** Transmittance spectrum, and **f** CLSM image of SiC transmission gratings. **g** Cross-sectional SEM image, **h** EDS distribution maps, and **I** HRTEM image of the internal SiC structure.

**Laser****-steering characteristics.** The laser-steering capability of the proposed design was next characterized through the use of dynamic 1D and 2D cascaded reconfigurable internal SiC transmission gratings. These gratings enable precise control of the laser beam direction through the adjustment of the diffraction properties in response to mechanical elongation. A single-diffraction principle is used in the 1D laser steering system, in which an incident laser beam interacts with one transmission grating (Fig. 3a). This process is governed by the equation dsinθ=mλ, where d is the grating period, θ is the diffraction angle, m is the diffraction order, and λ is the laser wavelength. Mechanical stretching dynamically alters the grating period, resulting in a tunable diffraction angle. A custom-designed square stretching device (Fig. S9), consisting of eight moving components clamping the grating edges and corners, ensures uniform elongation across the grating. These components were synchronously stretched along straight grooves. Optical microscopy images reveal that the grating period increases from approximately 10 µm to 13.8 µm under 38% elongation and fully recovers to its original value upon stress release (Fig. 3b). Furthermore, the embedded SiC gratings exhibit no visible cracking or delamination after repeated stretching cycles (up to 1000 times at 38% strain), demonstrating good mechanical stability for reconfigurable optical applications (Fig. S10). This tunable and reversible period enables controlled laser beam steering. Using a commercial 1550 nm distributed-feedback (DFB) laser and an infrared CCD camera, the movement of diffraction spots on a screen was recorded, which confirms the dynamic steering capability of the proposed system (Fig. 3c).

The 1D laser steering performance was evaluated in terms of the steering angle, diffraction efficiency, and beam quality (Figs. 3d-f). A linear relationship between the elongation angle and the steering angle was observed (Fig. 3d), with the steering angle of the ±5^th^-order beams reaching ~299 mrad at 38% elongation. The diffraction efficiency was also investigated by monitoring the variations in the intensities of beams with different diffraction orders (Fig. 3e). The changes in duty cycle affect the Fourier coefficients of the dielectric constant distribution in the grating region, leading to alterations in the energy distribution of diffracted light spots. When the SiC grating undergoes stretching, the Poisson's ratio mismatch between SiC grating fringes and the PDMS substrate causes smaller deformation in SiC fringes compared to the PDMS substrate. Consequently, the duty cycle decreases under stretching. Therefore, the intensity of the m=0 diffraction order decreases after 10% elongation, while the energy of other diffraction orders relatively increases. Furthermore, the beam quality of diffraction spots remains excellent during stretching and recovery. The beam profiles retain their Gaussian shape, and the energy distribution remains both uniform and symmetric (Fig. 3f). The M² factor of the diffracted beams is consistently less than 1.35, indicating high beam quality. In contrast, surface gratings under similar tensile conditions exhibit random degradation, with reduced beam quality (M^2^ > 1.5), due to structural damage (Fig. S11), which adversely affects scanning and transmission. Continuous-wave laser pyrolysis has recently enabled elegant and precise patterning of SiC microstructures on PDMS surfaces ^41^. Here, femtosecond laser pulses are used to generate embedded SiC transmission gratings, which maintain beam quality during stretching and enable dynamic beam steering essential for FSOC.

To enable 2D laser steering, a cascaded diffraction grating system was developed (Fig. 3g). This system is composed of two orthogonal 1D gratings that are independently tunable, allowing dynamic control of the laser beam in both the X and Y directions. The 2D system operates by synchronizing the stretching of both gratings, altering the diffraction direction in their respective planes. This approach effectively increases the steering coverage and the node capacity in point-to-multipoint FSOC systems. Additionally, the system has versatile characteristics for complex communication networks requiring dynamic steering in multiple directions. As shown in Fig. 3h, diffraction spots are observed in the array when the laser beam passes through the cascaded diffraction gratings. Stretching one grating results in the spot shifting along the corresponding axis, whereas stretching the other grating results in the spot shifting along the orthogonal axis. For example, the elongation of grating 1 results in the spot shifting along the Y-axis, whereas the elongation of grating 2 results in the spot shifting along the X-axis. The system's re-routing speed is determined by the reconfiguration rate of the SiC grating, which can be dynamically controlled by adjusting the rotational speed of the servo motor driving the square-stretching device. Consequently, for the 2D scanning system, the re-routing speeds in the X and Y directions can be independently tuned.

The laser steering performance of the cascaded array was also characterized on the basis of the changes in the steering angle, diffraction efficiency, and beam quality with mechanical elongation. The steering angle linearly increases with the increase of the elongation in both directions, reaching a maximum value of ~47.8 mrad at 38% elongation (Fig. 3i). The diffraction intensities for the (0^th^, +1^st^)-, (+1^st^, 0^th^)-, and (+1^st^, +1^st^)-order beams depend on the elongation amount, and the intensity variations were fitted to a predictive surface model (R^2^ > 0.9) (Fig. 3j and Fig. S12). The transmittance of 2D system remains >88.5% across 1530-1570 nm and the attenuation of each order beam is <2% within 0-35% elongation (Fig. S13). Similar to the 1D system, the beam quality is excellent in the cascaded system. The M² factor remains below 1.35, and the diffracted beams retain their Gaussian profile, ensuring high-quality laser steering in both dimensions (Fig. 3k). In contrast, under identical tensile conditions, the use of surface gratings results in the degradation of beam quality (M² > 5) compared with the use of the proposed internal SiC gratings (Fig. S14). In both the 1D and 2D steering experiments, the high beam quality and stable diffraction performance are attributed to the internal SiC grating structure and the use of the square stretching/recovery method. The internal SiC grating resists distortion caused by inconsistencies between the upper and lower surfaces during deformation, maintaining a planar configuration across various states. Additionally, the square stretching method mitigates fringe bending arising from biaxial stretching and ensures that the proportional geometric dimensions are preserved in the irradiated area. Owing to this stability, a constant angle and phase relationship is maintained between the incident beam and the grating. The proportional edge variation expands the effective area of the grating, further enhancing the flexibility and scalability of the transmitter. These data indicate that dynamic laser steering can be achieved via elongation. The diffraction efficiency and polarization evolution of 2D scanning systems are both independent of the polarization state of the incident laser (Fig. S15 and S16). This is mainly determined by two aspects. Firstly, the PDMS substrate used in the experiment and the generated SiC grid fringes can be approximately considered isotropic. Secondly, the SiC fringes and grating periods are both in the micrometer scale, effectively avoiding the influence of nanostructures on polarization states.

**
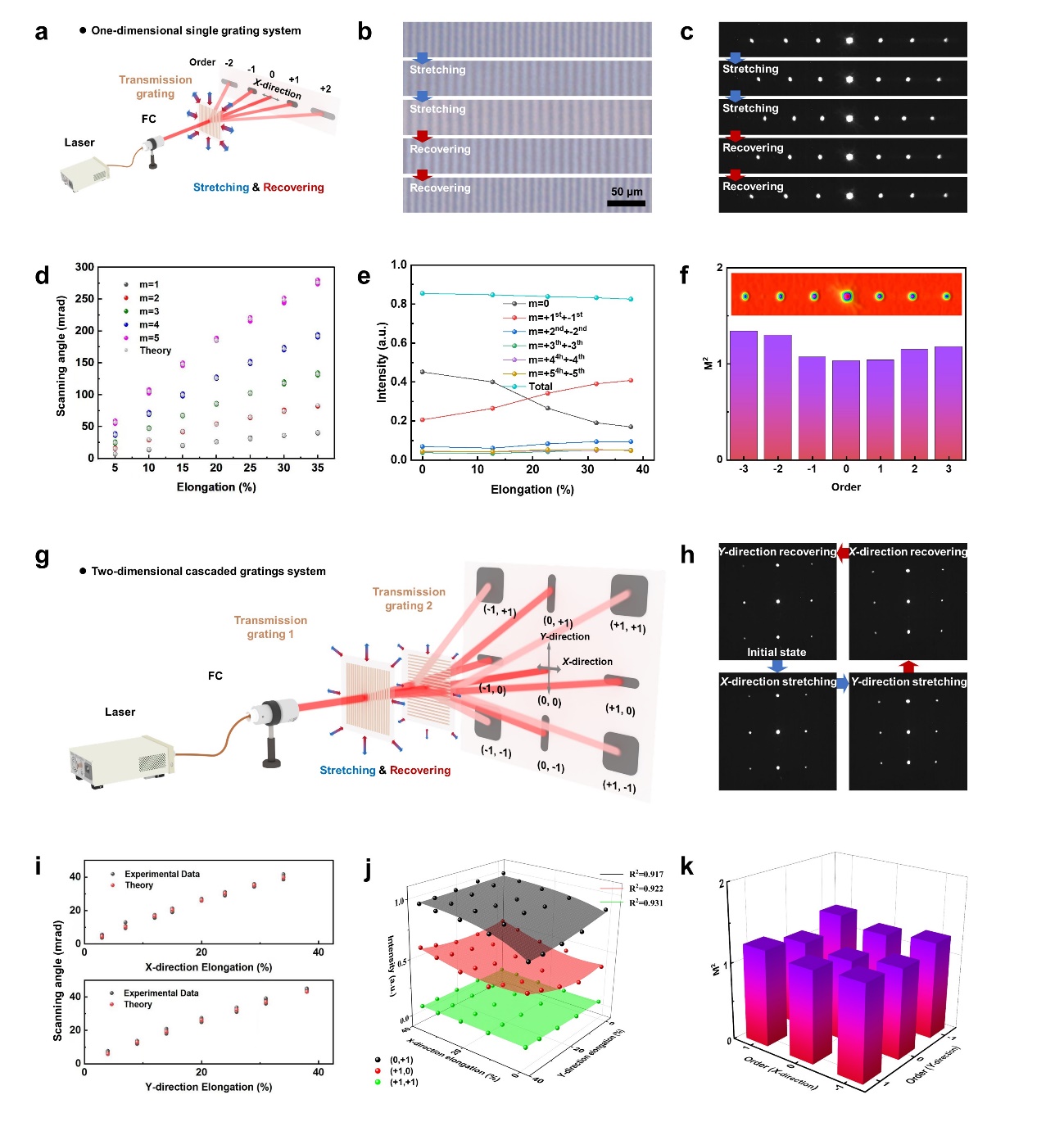
**

**Fig. 3. Laser steering characteristic****s of the stretchable internal SiC transmission gratings. a** Schematic illustration of the laser steering mechanism in the 1D configuration. **b** Optical images of the stretchable internal SiC transmission gratings and **c** Dark-field images showing 1D diffraction spots during the stretching and recovery processes. Dependence of the **d** steering angle and **e** diffraction efficiency on mechanical elongation in the 1D configuration. **f** Quality of the diffracted beams in the 1D configuration, with the inset showing the light field distributions of the diffraction spots. **g** Schematic representation of the laser steering mechanism in the cascaded 2D configuration. **h** Dark-field images showing the diffraction spots during stretching and recovery in the 2D configuration. Dependence of the **i** steering angle and **j** Diffraction efficiency on mechanical elongation in the cascaded 2D configuration. **k** Quality of the diffracted beams in the cascaded 2D configuration.

**Point-to-multipoint FSOC construction****.** The transmission characteristics of the point-to-multipoint FSOC system in a simulated atmospheric channel were next evaluated. The experimental setup for the 1D point-to-multipoint FSOC system is illustrated in Fig. 4a. A modulated laser beam with a wavelength of 1550 nm, carrying a 10 Gbps signal with a pseudorandom binary sequence (PRBS) of 2^9^, was used as the light source. The laser polarization was adjusted with a polarization controller, and the modulation state was optimized by controlling the bias voltage applied to the electro-optic modulator. To compensate for insertion loss during modulation, the optical power was amplified through a collimator before the beam moved from the fiber to free-space transmission. After passing through the stretchable internal SiC transmission grating, the beam was diffracted into multiple beams, which were transmitted through controllable atmospheric channels, and the system performance was evaluated.

In the simulated atmospheric channel, various turbulence intensities were simulated by creating convection between a cooling plate and a heating plate. The turbulence intensity was controlled by adjusting the temperature difference (ΔT) between the plates, with the atmospheric coherence length (r_0_) inversely proportional to ΔT. The relationship between r_0_ and ΔT is expressed by the following equation:

 (1)

Thus, the turbulence intensity increases as r_0​_ decreases. After being transmitted through the channel, the diffracted beams were collected with a collimator, converted back to fiber-based transmission beams, amplified, and analyzed. The signal quality was analyzed via a bit error rate (BER) tester and a wide-bandwidth oscilloscope (WBO).

Figs. 4b–d present the BER results for the 0 ^th^ to ±3 ^rd^-order diffraction beams under various turbulence intensities (r_0_>30 cm, 1.15 cm, and 0.66 cm) and an internal SiC transmission grating elongation of 38 %. As ΔT increases and r_0_ decreases, the BER increases across all the diffraction orders. As shown in the results, higher-order diffraction beams have greater BERs than lower-order diffraction beams. For instance, at r_0_​=0.66 cm and a received power of -18 dBm, the BER and signal-to-noise ratio (SNR) of the -3^rd^-order beam are 9.2×10^−8^ and 12.06 dB, respectively.

To extend the system into two dimensions, a cascaded 2D point-to-multipoint FSOC setup was developed (Fig. 4e). In this configuration, two perpendicular internal SiC transmission gratings were employed to achieve independent control of the diffraction beams in the X- and Y- directions. When the laser beam passes through the cascaded gratings, the diffraction spots move along the X-axis when the first grating is stretched and move along the Y-axis when the second grating is stretched (Figs. 4f–h). The BERs for the (0^th^, 0^th^)-, (0^th^, +1^st^)-, (+1^st^, 0^th^)-, and (+1^st^, +1^st^)-order diffraction beams were measured under various turbulence intensities (r_0_>30 cm, 1.15 cm, and 0.66 cm) with a grating elongation of 37.8%. Similar to the 1D system, the BER increases with increasing turbulence intensity, and higher-order diffraction beams have higher BERs than lower-order beams. For example, at r_0_=0.66 cm and a received power of -17 dBm, the BER and SNR of the (+1^st^, +1^st^)-order beam are 7.3×10^−7^ and 10.23 dB, respectively.

These experimental results highlight that, despite a slight reduction in the transmission capacity for higher-order beams, both 1D and 2D high-order diffracted beams exhibit substantial resistance to atmospheric turbulence despite significant grating elongation. It should be noted that, to achieve long-distance atmospheric transmission during multibeam routing, it is crucial to maintain the SiC grating's stretching ratio below a safety threshold. This safety value is intrinsically linked to the grating's structural characteristics, with the designed SiC grating in this work having a safe stretching ratio of 38%. Exceeding this limit may cause non-uniform deformation of the grating, leading to grating lobes in the diffracted optical field and resulting in additional losses. This robustness underscores the potential application of these gratings in FSOC networks in outdoor environments, enabling extended transmission distances and stable performance under dynamic atmospheric conditions.


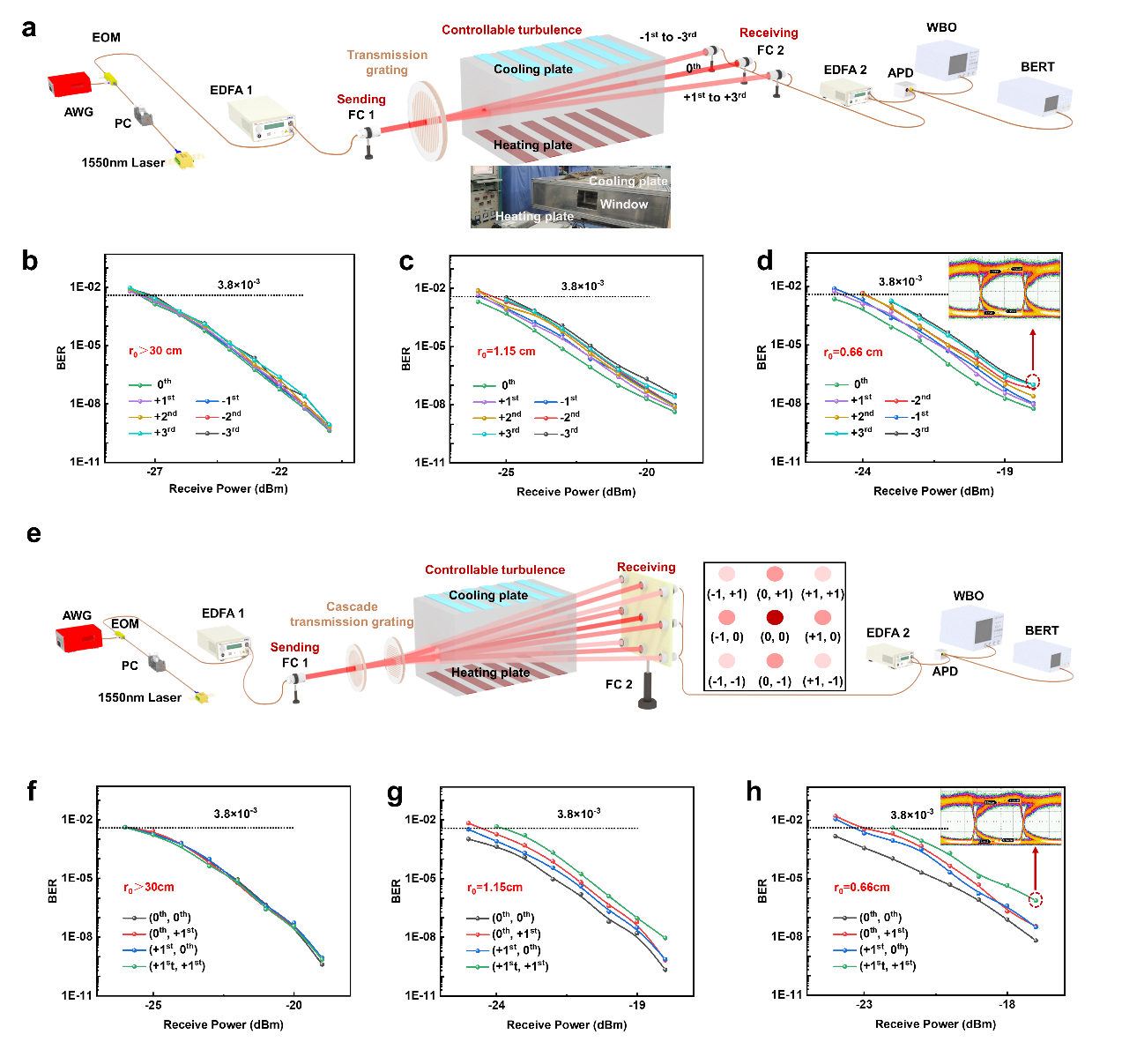


**Fig. 4. Transmission characteristics of the point-to-multipoint FSOC system in a controllable atmospheric channel. a** Schematic of the experimental setup for the 1D FSOC system. BER measurement for the 1D FSOC system **b** without turbulence and under **c** weak turbulence and **d** strong turbulence. **e** Schematic of the experimental setup for the cascaded 2D FSOC system. BER measurement for the cascaded 2D FSOC system **f** without turbulence and under **g** weak turbulence and **h** strong turbulence.

**Outdoor point-to-multipoint atmospheric FSOC experiments****.** Outdoor experiments were next conducted to evaluate the performance of the point-to-multipoint FSOC system. A schematic diagram of the 1D 1-to-7 link transmission system was presented in Fig. 5a. The experimental setup spanned a 225-meter real atmospheric link between two buildings (A and B), with the laser transmission window located on building A (Fig. 5b). The transmitting end consisted of a modulated laser source, a transmission grating, an erbium-doped fiber amplifier, and an alignment system (Fig. 5c). A PRBS was modulated onto a 1550 nm laser beam, which was then amplified by the erbium-doped fiber amplifier. The amplified beam passed through the transmission grating, generating a 1D diffracted beam directed toward the receiving end on building B (Fig. 5d). The steering angle ranges for beams of different diffraction orders were determined by varying the elongation of the internal SiC transmission gratings from 0% to 37.8%. For the ±1^st^, ±2^nd^, and ±3^rd^-order beams, the steering angle ranges were 12.9 mrad, 26 mrad, and 39.1 mrad, respectively. BER measurements for the 0^th^ to ±3^rd^-order beams are shown in Figs. 5e–g for elongations of 0%, 22.7%, and 37.8%, respectively. The results indicate that as the elongation increases, the BERs of higher-order beams at the same received power increase, demonstrating that these beams are more sensitive to stretching than are lower-order beams. For example, at 37.8% elongation and a received power of -17 dBm, the BER and SNR for the -3rd-order beam are 5.83 × 10⁻⁷ and 5.57 dB, respectively.

The system was further extended to a 2D 1-to-9 FSOC transmission system (Fig. 5h). In this setup, the cascaded transmission gratings generate and transmit 2D diffracted beams. These beams are then captured at the receiving end (Fig. 5i). Secondary diffraction through the cascaded gratings enabled the (±1^st^, ±1^st^)-order beam to be freely scanned within a rectangular area in the 2D X‒Y plane by tuning the elongation of each grating. At 37.8 % elongation, the steering angle range in both the X and Y directions was 12.9 mrad. In addition to PRBS data transmission, the system successfully transmitted video content. Fig. 5j_1_ and Fig. S17 display snapshots at different timestamps (01:49:00, 10:10:00, and 16:40:00) carried by the (0^th^, 0^th^)-, (+1^st^, 0^th^)-, and (+1^st^, +1^st^)-order beams. The corresponding audio signals are shown in Fig. 5j_2_. The received image and audio quality are consistent across beams of all tested orders, demonstrating the reliable data transmission capability of the proposed system. BER measurements for the (0^th^, 0^th^)-, (0^th^, +1^st^)-, (+1^st^, 0^th^)-, and (+1^st^, +1^st^)-order beams are provided as representative examples (Figs. 5k-n). Similar trends are observed in the 2D link system compared with those in the 1D link system, with the BERs of higher-order beams increasing as the grating elongation increases. At 37.8% elongation and a received power of -16 dBm, the BER and SNR for the (+1^st^, +1^st^)-order beam are 2.24 × 10^-7^ and 4.97 dB, respectively. Owing to the limited area of building B, only lower-order diffracted beams are demonstrated to ensure that all receiving nodes remain within the range of the system. However, in larger spaces, higher-order beams can be utilized to increase the number of transmission nodes and the dynamic range of the system. The system is characterized by its lightweight and flexible design, which makes it suitable for use on low-load platforms such as small unmanned aerial vehicles.


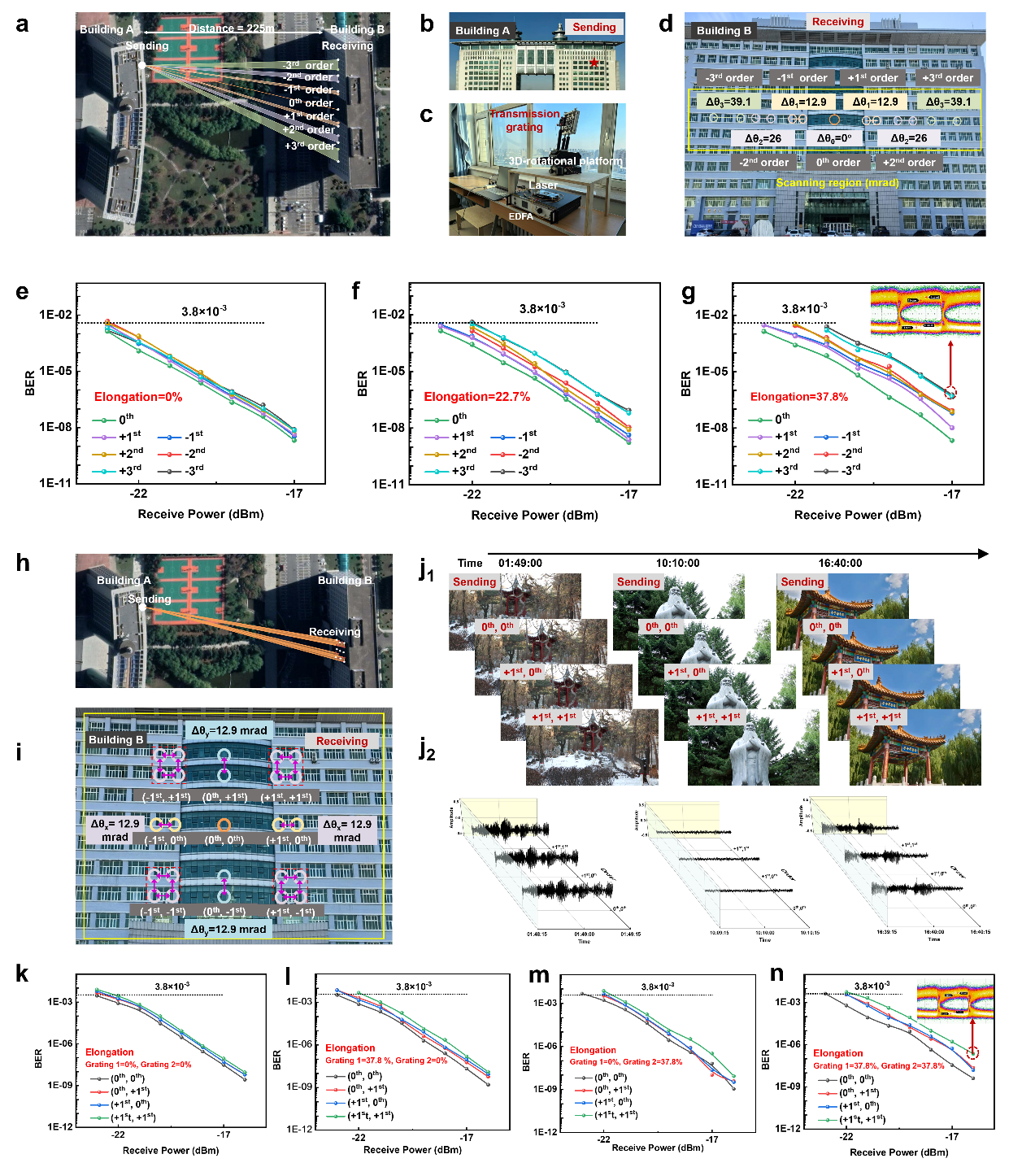


**Fig. 5.** **Point-to-multipoint outdoor FSOC system over a 225-meter real atmospheric channel.** **a** Diagram of the 1D 1-to-7 FSOC link scenario. **b** Laser transmitting window. **c** Experimental configuration of the sending end. **d** Receiving nodes for the 0^th^ ±3^rd^-order beams. **e, f, and g** BER measurements of the 1D 1-to-7 FSOC link under 0% elongation, 22.7% elongation, and 37.8% elongation, respectively. **h** Diagram of the cascaded 2D 1-to-9 (3×3) FSOC link scenario. **i** Receiving nodes for the (0^th^, 0^th^)- to (±1^st^, ±1^st^)-order beams. **j** Video transmitted using the (+1^st^, +1^st^)-order beam. **k-n** BER measurements of the 2D 1-to-9 (3×3) FSOC link under different grating elongations: **k** grating 1=0%, grating 2=0%; **l** grating 1=37.8%, grating 2=0%; **m** grating 1=0%, grating 2=37.8%; and **n** grating 1=37.8%, grating 2=37.8%.

**Discussion**

This paper introduces a groundbreaking approach to develop portable point‒to‒multipoint FSOC networks by leveraging reconfigurable SiC transmission gratings directly formed in stretchable PDMS films. These gratings are fabricated using femtosecond laser technology, and innovative optical, mechanical, and material techniques are used to create a system with high transmittance (~91.9% at 1550 nm), dynamic beam steering capability (up to ~299 mrad for 1D systems and ~47.8 mrad for cascaded 2D systems), and exceptional beam quality. Specifically, at 37.8% elongation and -16 dBm power, the BER is 5.83 × 10⁻⁷ for the -3^rd^-order beam in the 1D configuration and 2.24 × 10^-7^ for the (+1^st^, +1^st^)-order beam in the 2D configuration, underscoring the scalability and adaptability of the proposed gratings to diverse environmental conditions. In contrast to rigid SiC gratings fabricated by lithography and etching on inflexible substrates ^42-44^, the embedded and reconfigurable gratings developed in this work enable strain-driven beam steering, providing dynamic tunability that is critical for point-to-multipoint optical communication. These state-of-the-art capabilities are validated in an outdoor experiment involving a 225-meter transmission distance, in which the system reliably transmits Gbps-level data with low BERs, even under elongation and atmospheric turbulence. Importantly, compared with existing metal micro/nanodevices^12, 19, 20, 22, 45-47^, our scheme significantly increases the transmission distance by two orders of magnitude while also incorporating functionalities such as beam manipulation and multinode communication (Extended Data Table 1). As our solution uses diffraction-based beam steering for multiple optical beams, the beam divergence angle depends on both the diffraction order and the grating elongation. Consequently, the evaluation of transmitter and spatial losses in our approach differs fundamentally from that in benchmark fixed-lens systems. We also provide a detailed comparison of atmospheric optical communication link penalty evaluation methodologies between our proposed scheme and benchmark lenses in Extended Data Table 2, offering critical references for subsequent link design. Our scheme is underpinned by the lightweight and compact design of the system, which eliminates the need for bulky mechanical steering components, which are commonly used in conventional FSOC systems.

Future work should focus on further enhancing the capabilities of the system. For example, the diffraction efficiency at higher orders can be increased through the use of an incident laser with higher power or a refined grating design. The dynamic range can be optimized by tailoring the SiC grating period to align with the motion characteristics of the receiving targets. To extend the transmission distance, a laser with higher power, error correction algorithms and adaptive optics techniques could be used to mitigate atmospheric attenuation. Additionally, the transmission rate could be significantly increased through the use of bandwidth optimization or multiplexing techniques.

This work establishes a strong foundation for the development of next-generation FSOC networks, offering a lightweight, tunable design with advantages such as flexibility, scalability, and high-speed performance. Its compact form factor makes it ideal for use on small-scale platforms such as microsatellites, unmanned aerial vehicles, and compact ground stations, satisfying critical weight and space constraints. The developed approach has substantial potential in space–ground and urban infrastructure applications and paves the way for innovations in long-range, high-capacity atmospheric optical communication networks.

**Materials and methods**

**Fabrication of the reconfigurable internal SiC gratings.** PDMS (Sylgard 184B, Dow Corning) was used. First, an electronic balance was employed to weigh the materials, maintaining a PDMS-to-curing agent ratio of 10:1. To achieve uniform mixing and remove air bubbles, the mixture was sealed in a test tube and centrifuged at 4000 rpm for 10 minutes. The resulting mixture was evenly poured onto a 3 cm × 3 cm × 0.3 cm glass slide. The sample was then cured in an electrothermal dryer at 70°C for 1 hour, producing a PDMS film with an area of 3 cm × 3 cm and a thickness of 0.893 mm. A femtosecond laser direct writing technique was subsequently employed to fabricate a SiC grating line structure within the PDMS film. The femtosecond laser used for SiC grating formation was a Pharos system (Light Conversion), operating at a central wavelength of 1030 nm, with a pulse duration of 280 fs and a repetition rate of 200 kHz. The average output power was maintained at approximately 100 mW, corresponding to a pulse energy of ~0.5 μJ. Laser pulses were focused into the PDMS substrate using a 10× objective lens (NA = 0.25), producing a focal spot radius of approximately 5 μm. The peak intensity per pulse was estimated using the equation I = 2E/(πr^2^τ), where E is the pulse energy, r is the focal spot radius, and τ is the pulse duration. The resulting peak intensity is approximately 2.84×10^12^ W·cm^-2^, sufficient to induce nonlinear absorption and localized SiC formation within the PDMS matrix. The scanning speed is 5 mm·s^-1^. A 10 mm × 10 mm area was processed with a line-to-line spacing of 10 μm.

**Material characterization.** Photographs were taken using an Olympus TG-3 camera. Transmittance spectra were measured with a Shimadzu UV-3600 spectrophotometer. CLSM images were obtained using a 3D measuring laser microscope (LEXT, Olympus, OLS4100). SEM images were acquired with a JEOL JSM-7500 field-emission scanning electron microscope. HRTEM images were captured using an FEI Tecnai G2 F20 transmission electron microscope.

**Molecular dynamics simulations.** In the MD simulations, a box with dimensions of approximately 50 × 50 × 50 Å^3^ was created. Then, 10 PDMS polymer chains, each composed of 100 monomers, were randomly placed into the box. As a result, the system consisted of 2000 carbon, 6010 hydrogen, 1010 oxygen and 1000 silicon atoms. Periodic boundary conditions (PBCs) were imposed in three orthogonal directions to mimic the boundary conditions of large structures. To describe the atomic interactions in the PDMS system, the reactive force field (ReaxFF) potential [https://doi.org/10.1021/jp306391p] was utilized. Prior to the MD simulations, the as-constructed PDMS system was optimized according to a local configuration with energy and force tolerances of 1.0 × 10^-2^ kcal·mol^-1^ and 1.0 × 10^-2^ kcal·(mol·Å)^-1^, respectively. Then, MD simulations were performed to further relax the samples. The simulations were performed with 400000 timesteps at a temperature of 300 K for the thermostatic layer under NVT (constant number of particles, constant volume, and constant temperature) conditions, and the temperature was controlled with a nose-hoover thermostat. Subsequently, atoms in a spherical region with a radius of 10 Å in the center of the as-created PDMS were heated to 5000 K for 40000000 timesteps to investigate the structural changes. Finally, the as-heated PDMS system was cooled to 300 K. To better reflect real-world experimental conditions, in which gas molecules escape from the irradiated region, small molecules such as H_2_O, H_2_, CO_2_, and CO generated during the laser heating process were artificially removed in the simulation. The motions of the atoms in the PDMS system followed Newton’s motion, and the velocity-Verlet algorithm with a timestep of 0.25 fs was applied to integrate Newton’s equations. All the MD calculations were implemented using the Large-scale Atomic-Molecular Massively Parallel Simulator (LAMMPS) software package [https://doi.org/10.1006/jcph.1995.1039].

**Laser steering characterization.** Dark-field images were captured using a 1550 nm CCD camera (Gatherstar, GS-SW6401715-UC-CL). The powers of the 1D and 2D diffracted beams were measured with an optical power meter (Thorlabs, S145C). The quality of the 1D and 2D diffracted beams was assessed using an M² measurement system (Thorlabs, M2MS-BP209IR2).

**Point-to-multipoint FSOC experiments.** Fig. 4a shows a schematic diagram of the 1D steering system tested in an atmospheric transmission channel. A DFB laser source (CONQUER, KG-DFB9002) operating at 1550 nm served as the carrier wave, which was modulated by an electro-optic modulator (EOM, Oclaro, 7910553-B) to encode information. A polarization controller (PC) was used to adjust the laser polarization to optimize the modulation performance of the system. An arbitrary waveform generator (Keysight, M8195A, 65 GSa·s^-1^) was used to produce a 10 Gbps signal with a voltage of 500 mVpp. To mitigate the insertion loss caused by the modulator, an erbium-doped fiber amplifier (EDFA1, Connet, MFAS-EY-C-B) was used to amplify the optical signal to 20 dBm. A fiber collimator (FC1, F810FC-1550) was used to change the transmission of the collimated laser beam from the fiber to free space. As the laser passed through the PDMS grating, multiple diffracted beams were generated, each carrying digital signals and directed in different directions. These beams propagated through a controllable turbulence channel, and the signal quality under various turbulence intensities was evaluated. The diffracted beams were collected with fiber collimators (FC2, F810FC-1550) and transmitted back to the optical fibers. A secondary erbium-doped fiber amplifier (EDFA2, Connet, MFAS-1550-B-FA) was used to amplify the weak signals, which were detected with an avalanche photodiode (APD, CONQUER, APD-10G22482). Finally, a BER tester (BERT, N10000A, 10 Gbps) and a WBO (Agilent, 86100C) were employed to measure the BER and SNR under clock-synchronized conditions.

Fig. 4e presents a schematic diagram of the 2D steering system tested over an atmospheric transmission channel. In the transmitting and receiving setups, the same devices as those employed in the 1D steering and information transmission system were utilized. In contrast to the 1D configuration in this experiment, two cascaded SiC gratings with perpendicular orientations were incorporated, which functioned as steering and beam-splitting elements. When the laser beam passed through the cascaded gratings, a 2D array of diffracted beams was generated. The movement of these beams within the 2D space was precisely controlled by independently stretching the two gratings along their respective axes. This configuration facilitates dynamic and flexible 2D beam steering, providing increased control and adaptability for FSOC systems.

At the transmitting end, a PRBS signal and a modulated beam were generated and output by a Kintex-7-based field-programmable gate array (FPGA) development board (XC7K325T-2FFG676I). The SNR of the signal carried by the diffracted beams was measured using a WBO, whereas the BER was assessed using the same FPGA development board. For the outdoor experiment, the signal was output by the Kintex-7-based FPGA development board, and the modulated beam was produced by an optical module (OSX080N03), as shown in Fig. S18. The PRBS generated by the FPGA development board had a length of 215, and the transmission rate was 10 Gbps. Fig. S19 depicts the optical transceiver employed to transmit a 936 MB video, as illustrated in Fig. 5j. The video was transmitted from a computer connected to the RJ45 socket of the transceiver, where it was encoded by the internal encoding driver of the transceiver. The encoded information was then modulated onto the laser beam via a modulation driver. The same optical transceiver as that employed at the transmitting end was used at the receiving end. Fig. S20 shows the optical receiving device used at the receiving end, which is a custom-built theodolite-type FSOC terminal designed for small platforms. The received beam was captured by a telescope system and coupled into the optical transceiver.

**Experimental method for video transmission.** An optical transceiver was used to transmit a 936 MB video, as shown in Fig. 5j. Fig. S20 presents the structure of the optical transceiver and a schematic diagram of the transmission process. The transceiver had a maximum transmission rate of 1 Gbps and operated at a wavelength of 1553.96 nm. The video was sent from a computer connected to the RJ45 socket of the transceiver, where it was encoded by the internal encoding driver of the transceiver. Then, the encoded information was modulated onto the laser beam by the modulation driver. The same optical transceiver as was used at the transmitting end was employed at the receiving end. The diffracted beam carrying the signal was coupled into a fiber using a fiber collimator and transmitted to the second optical transceiver, which had a minimum received power threshold of -25 dBm. Upon entering the transceiver, the light beam was converted into an electrical signal by a PIN or an APD detector and subsequently amplified by a transimpedance amplifier (TIA). The signal was then processed through a low-pass (LP) and an RF amplitude limiter (RF AMP limiter) before being further amplified by a line driver. The amplified electrical signal was demodulated by the demodulator and decoded by the internal decoding module of the transceiver. Finally, the decoded signal was transmitted to another computer connected to the receiving transceiver via the RJ45 socket.

**Data availability**

All the data supporting the findings of this study are available within this Article and its Supplementary Information. Any additional information can be obtained from the corresponding authors on reasonable request.

**Acknowledgements**

This work was supported by the National Natural Science Foundation of China (U2141231, 62275100, T2325014)； Natural Science Foundation of Jilin Province (20240101020JJ); Natural Science Foundation of Chongqing Municipality (CSTB2023NSCQ-MSX0030).

**Conflict of interest**

The authors declare no conflict of interest.

**Contributions**

W.M. and D.H. conceived the concept. W.M., Y.F., D.H., J.Z., Q. W., and Y.Z., designed and fabricated the reconfigurable internal SiC gratings. W.M., Y.F., K.D., J.Z., P.L., Z.L., Y. G., X. L and H.J. and characterized the laser steering characteristic and carried out outdoor point-to-multipoint FSOC experiments. All the authors discussed the results and contributed to the writing of the paper.

**References**

1. Zhou, Z. C. et al. Prospects and applications of on-chip lasers. *eLight* **3**, 2200027x (2023).
2. Yao, B. C. et al. Interdisciplinary advances in microcombs: bridging physics and information technology. *eLight* **4**, 24000719 (2024).
3. Wang, W. T. et al. Free-space terabit/s coherent optical links via platicon frequency microcombs. *eLight* **5**, 25000820 (2025).
4. Heine, F. et al. Optical inter-satellite communication operational. 2010 - MILCOM 2010 Military Communications Conference*.* San Jose, CA, USA: IEEE, 2010, 1583-1587.
5. Liao, S. K. et al. Satellite-to-ground quantum key distribution. *Nature* **549**, 43-47 (2017).
6. Li, L. et al. Experimental demonstration of beaconless beam displacement tracking for an orbital angular momentum multiplexed free-space optical link. *Optics Letters* **43**, 2392-2395 (2018).
7. Wang, X. et al. On-orbit space optical communication demonstration with a 22 s acquisition time. *Optics Letters* **48**, 5980-5983 (2023).
8. Yu, X. N. et al. 2.5 Gbps free-space optical transmission between two 5G airship floating base stations at a distance of 12 km. *Optics Letters* **46**, 2156-2159 (2021).
9. Goldman, D. A. et al. MOEMS-based lens-assisted beam steering for free-space optical communications. *Journal of Lightwave Technology* **41**, 2675-2690 (2023).
10. Lin, P. et al. Research on one-to-two-point FSO system based on liquid crystal variable retarder cascade polarization grating. *Optics Express* **31**, 44693-44702 (2023).
11. Peng, T. et al. Multi-target free-space laser communication system based on a rotating double prism with an RF signal beacon. *Optics Express* **30**, 22221-22232 (2022).
12. Han, R. L. et al. Multi-dimensional and large-sized optical phased array for space laser communication. *Optics Express* **30**, 5026-5037 (2022).
13. Cheben, P. et al. Subwavelength integrated photonics. *Nature* **560**, 565-572 (2018).
14. Xu, H. N. et al. Meta-structured silicon nanophotonic polarization beam splitter with an optical bandwidth of 415 nm. *Laser & Photonics Reviews* **17**, 2200550 (2023).
15. Leng, B. R. et al. Meta-device: advanced manufacturing. *Light: Advanced Manufacturing* **5**, 117-132 (2024).
16. Huang, H. Q. et al. Leaky-wave metasurfaces for integrated photonics. *Nature Nanotechnology* **18**, 580-588 (2023).
17. Guo, X. X. et al. Molding free-space light with guided wave–driven metasurfaces. *Science Advances* **6**, eabb4142 (2020).
18. Ma, Q. et al. Smart metasurface with self-adaptively reprogrammable functions. *Light*: *Science & Applications* **8**, 98 (2019).
19. Huang, J. N. et al. A 20-Gbps beam-steered infrared wireless link enabled by a passively field-programmable metasurface. *Laser & Photonics Reviews* **15**, 2000266 (2021).
20. Wu, Y. et al. Tbps wide-field parallel optical wireless communications based on a metasurface beam splitter. *Nature Communications* **15**, 7744 (2024).
21. Hu, J. H. et al. A metasurface-based full-color circular auto-focusing Airy beam transmitter for stable high-speed underwater wireless optical communications. *Nature Communications* **15**, 2944 (2024).
22. Cao, Z. Z. et al. Reconfigurable beam system for non-line-of-sight free-space optical communication. *Light*: *Science & Applications* **8**, 69 (2019).
23. Yessenov, M. et al. Free-space optical delay line using space-time wave packets. *Nature Communications* **11**, 5782 (2020).
24. Mididoddi, C. K. et al. Ultrafast user localization and beam steering in optical wireless communication using an in-fibre diffraction grating. 2018 International Topical Meeting on Microwave Photonics (MWP)*.* Toulouse, France: IEEE, 2018, 1-4.
25. Shaboy, D., Rockban, D. & Handelman, A. Tapping underwater wireless optical communication in pure water and natural Dead-Sea ultra-high-salinity water by diffraction grating. *Optics Express* **26**, 29700-29711 (2018).
26. Lei, T. et al. Massive individual orbital angular momentum channels for multiplexing enabled by Dammann gratings. *Light*: *Science & Applications* **4**, e257 (2015).
27. Rola, K. P. et al. Focused electron beam micro- and nanopatterning of thin films derived from sol–gels based on TiO_2_ precursors for planar photonics. *ACS Applied Nano Materials* **7**, 8692-8702 (2024).
28. Peinado, P., Sangiao, S. & de Teresa, J. M. Focused electron and ion beam induced deposition on flexible and transparent polycarbonate substrates. *ACS Nano* **9**, 6139-6146 (2015).
29. Conrads, L. et al. Reconfigurable and polarization-dependent grating absorber for large-area emissivity control based on the plasmonic phase-change material In_3_SbTe_2_. *Advanced Optical Materials* **11**, 2202696 (2023).
30. Fan, Y. et al. Electric-driven flexible-roller nanoimprint lithography on the stress-sensitive warped wafer. *International Journal of Extreme Manufacturing* **5**, 035101 (2023).
31. Wu, K. M. et al. Highly flexible and electrically controlled grating enabled by polymer dispersed liquid crystal. *Journal of Molecular Liquids* **353**, 118664 (2022).
32. Liu, Y. et al. High-uniformity submicron gratings with tunable periods fabricated through femtosecond laser-assisted molding technology for deformation detection. *ACS Applied Materials & Interfaces* **14**, 16911-16919 (2022).
33. Kim, S. et al. Passive isothermal film with self-switchable radiative cooling-driven water sorption layer for arid climate applications. *Nature Communications* **15**, 8000 (2024).
34. Park, S. et al. Self-powered ultra-flexible electronics via nano-grating-patterned organic photovoltaics. *Nature* **561**, 516-521 (2018).
35. Kang, J. et al. Tough-interface-enabled stretchable electronics using non-stretchable polymer semiconductors and conductors. *Nature Nanotechnology* **17**, 1265-1271 (2022).
36. Lu, X. Y. et al. Emerging integrated laser technologies in the visible and short near-infrared regimes. *Nature Photonics* **18**, 1010-1023 (2024).
37. Yang, S. H. et al. Nonreciprocal thermal photonics. *Nature Photonics* **18**, 412-424 (2024).
38. Shayegan, K. J. et al. Direct observation of the violation of Kirchhoff’s law of thermal radiation. *Nature Photonics* **17**, 891-896 (2023).
39. Day, A. M. et al. Laser writing of spin defects in nanophotonic cavities. *Nature Materials* **22**, 696-702 (2023).
40. Nitiss, E. et al. Optically reconfigurable quasi-phase-matching in silicon nitride microresonators. *Nature Photonics* **16**, 134-141 (2022).

1. [Shin, J.](https://www.nature.com/articles/s41563-020-0769-6?fromPaywallRec=false#auth-Jaeho-Shin-Aff1-Aff2) et al. Monolithic digital patterning of polydimethylsiloxane with successive laser pyrolysis. [*Nature Materials*](https://www.nature.com/nmat) **20***,* 100-107 (2021).
2. Greffet, J. J. et al. Coherent emission of light by thermal sources. *Nature* **416**, 61-64 (2002).
3. Gubbin, C. R. et al. Strong and coherent coupling between localized and propagating phonon polaritons. *Physical Review Letters* **116**, 246402 (2016).
4. Folland, T. G. et al. Vibrational coupling to epsilon-near-zero waveguide modes. *ACS Photonics* **7**, 614-621 (2020).
5. Tao, J. et al. Beam-steering metadevices for intelligent optical wireless-broadcasting communications. *Advanced Photonics Research* **4**, 2300127 (2023).
6. Tao, J. et al. Beam-steering metasurfaces assisted coherent optical wireless multichannel communication system. *Nanophotonics* **12**, 3511-3518 (2023).
7. He, N. et al. High-speed duplex free space optical communication system assisted by a wide-field-of-view metalens. *ACS Photonics* **10**, 3052-3059 (2023).

**Additional information**

Supplementary Information is available for this paper.

Correspondence: Wanzhuo Ma (mawz@cust.edu.cn), Han Dongdong ([handongdong@jlu.edu.cn](mailto:handongdong@jlu.edu.cn)), Keyan Dong (dongkeyan@cust.edu.cn) and Yonglai Zhang (yonglaizhang@jlu.edu.cn).

Correspondence and requests for materials should be addressed to mawz@cust.edu.cn, handongdong@jlu.edu.cn, dongkeyan@cust.edu.cn or yonglaizhang@jlu.edu.cn.

The online version contains supplementary material available at https://doi.org/XXX.

Reprints and permissions information is available at www.nature.com/reprints.Supplementary information for

**Reconfigurable SiC gratings in PDMS: A portable approach for atmospheric optical communication networks**

Wanzhuo Ma^1, 3 #, *^, Yanwei Fu^1, 3 #^, Dong-Dong Han^2, *^, Keyan Dong^1, 3, *^, Jiaqing Zeng^1, 3^, Qiang Wang^2^, Peng Lin^1^, Yong-Lai Zhang^2, *^, Ye Gu^3^, Zhi Liu^1^, Xianzhu Liu^1^, Huilin Jiang^1^

^1^ Space Optoelectronic Technology Research Institute, Changchun University of Science and Technology, Changchun 130022, China

^2^ State Key Laboratory of Integrated Optoelectronics, Jilin University, Changchun 130012, China

^3^ College of Opto-Electronic Engineering, Changchun University of Science and Technology, Changchun 130022, China

^#^ These authors contributed equally: Wanzhuo Ma, Yanwei Fu.

E-mail: mawz@cust.edu.cn, handongdong@jlu.edu.cn, dongkeyan@cust.edu.cn, [yonglaizhang@jlu.edu.cn](mailto:yonglaizhang@jlu.edu.cn)

**Extended Data**


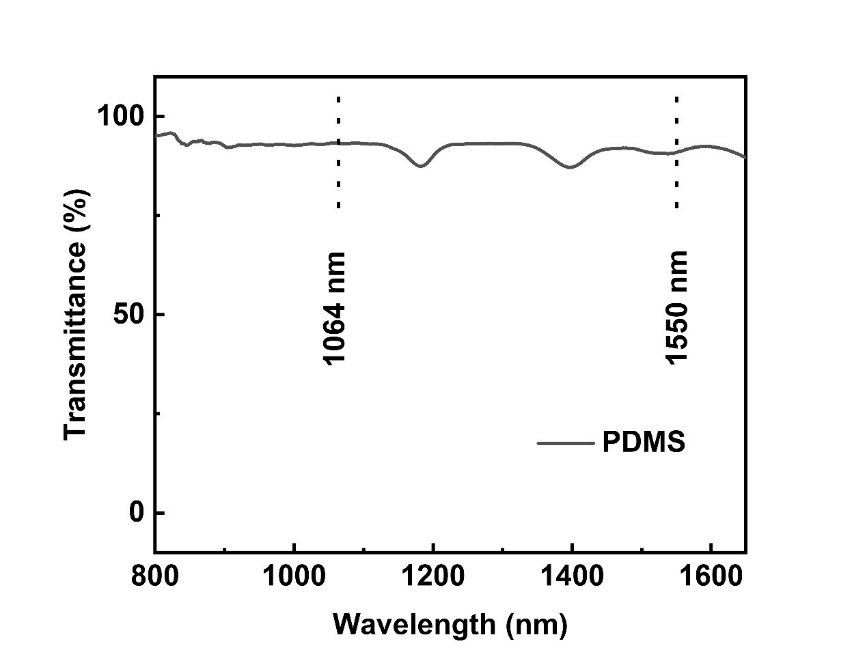


**Fig. S1** Transmittance spectrum of the PDMS film.


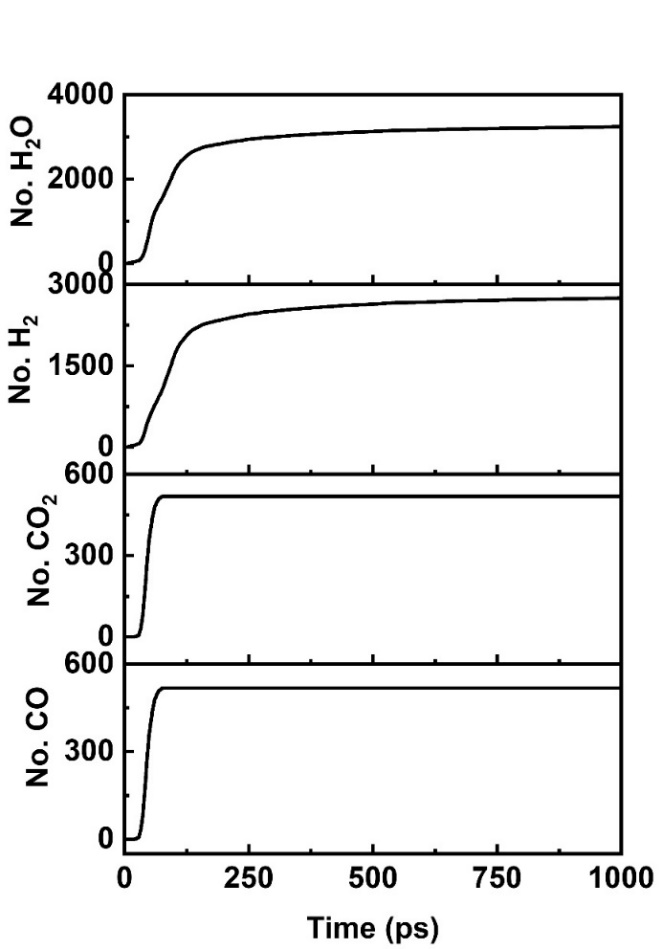


**Fig. S2** Variations in the number of H_2_O, H_2_, CO_2_ and CO molecules in the PDMS system with MD simulation time.


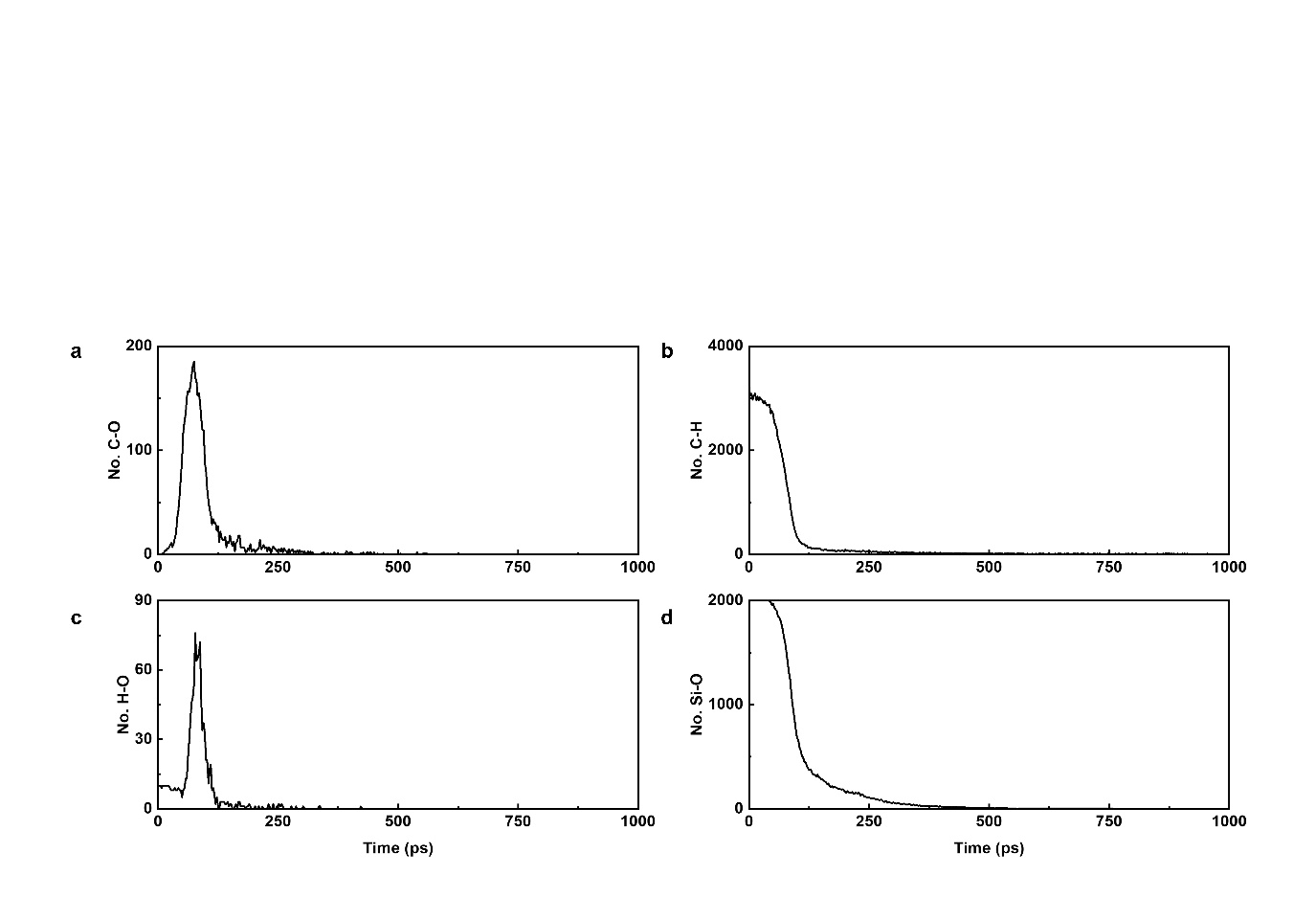


**Fig S3** Variation in the number of (a) C-O, (b) C-H, (c) H-O and (d) Si-O bonds in the PDMS system subjected to thermal-heating with MD simulation time.


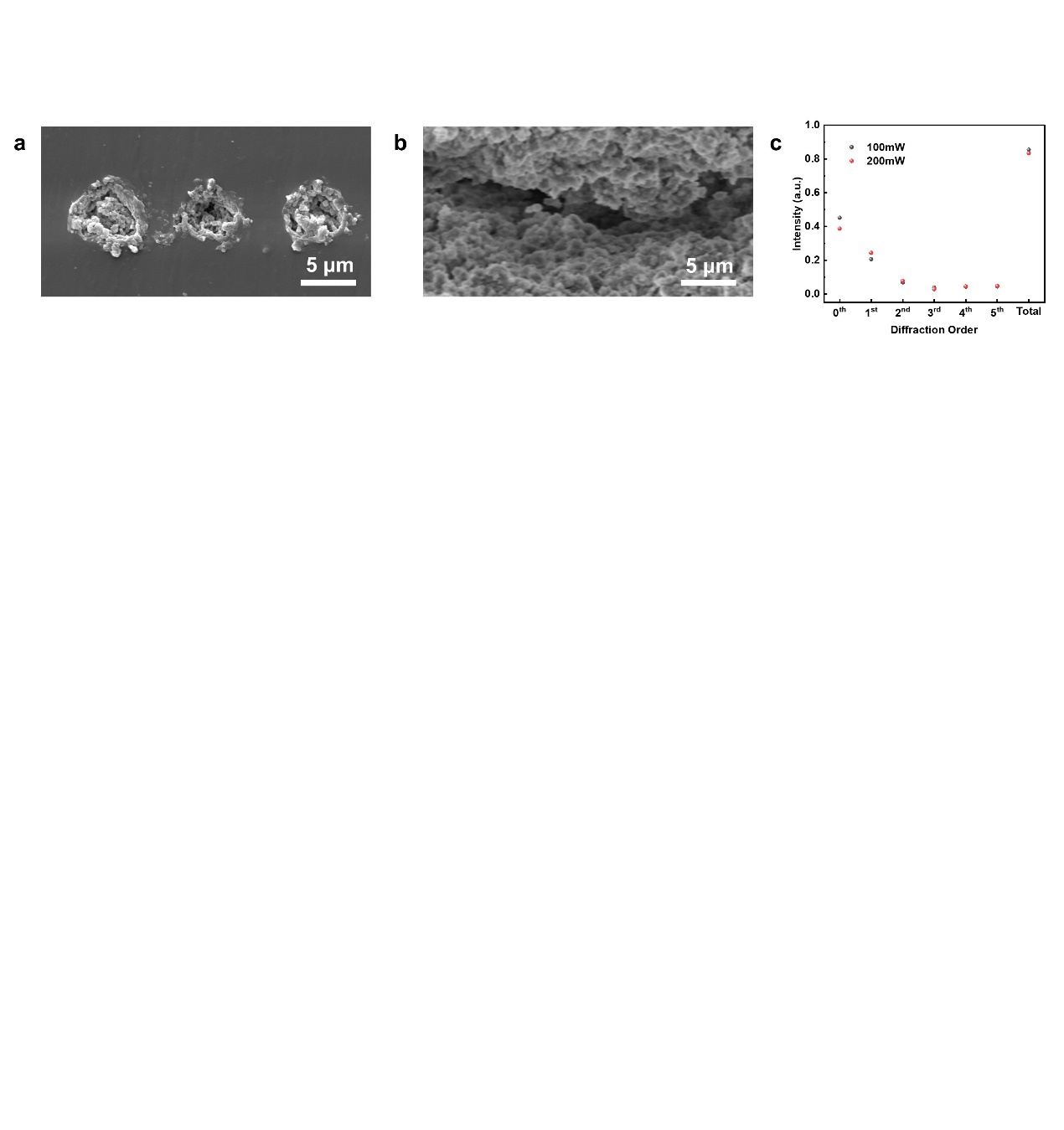


**Fig. S4** Cross-sectional SEM images of SiC gratings fabricated at pulse energies of (a) 1 μJ and (b) 2.5 μJ. The grating thickness increases with energy, while excessive energy leads to feature merging and loss of definition. (c) Intensity distribution of diffracted beams with different laser processing power.


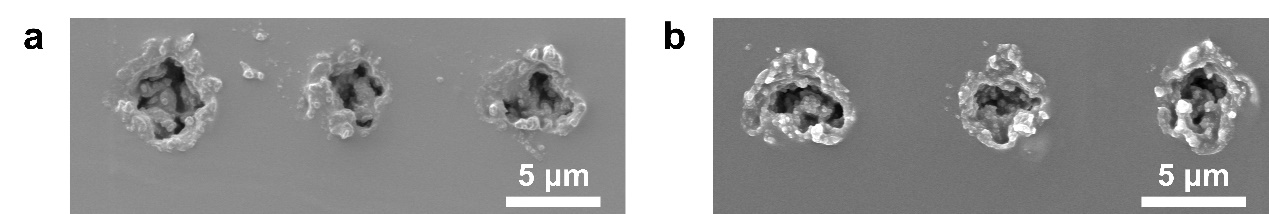


**Fig. S5** Cross-sectional SEM images of SiC gratings fabricated in PDMS with curing ratios of (a) 5:1 and (b) 20:1, showing similar feature sizes under identical laser conditions.


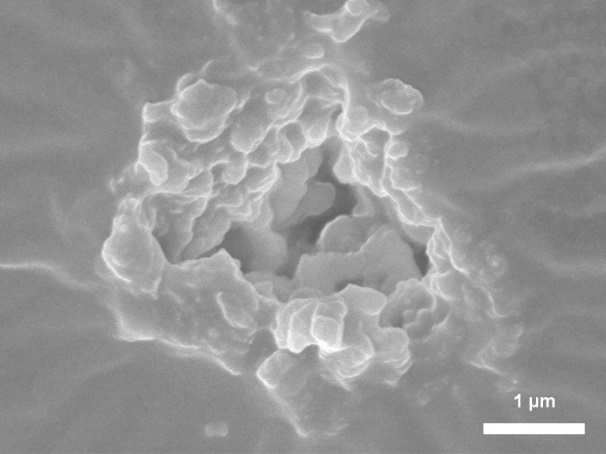


**Fig. S6** High-resolution cross-sectional SEM image of the grating line.


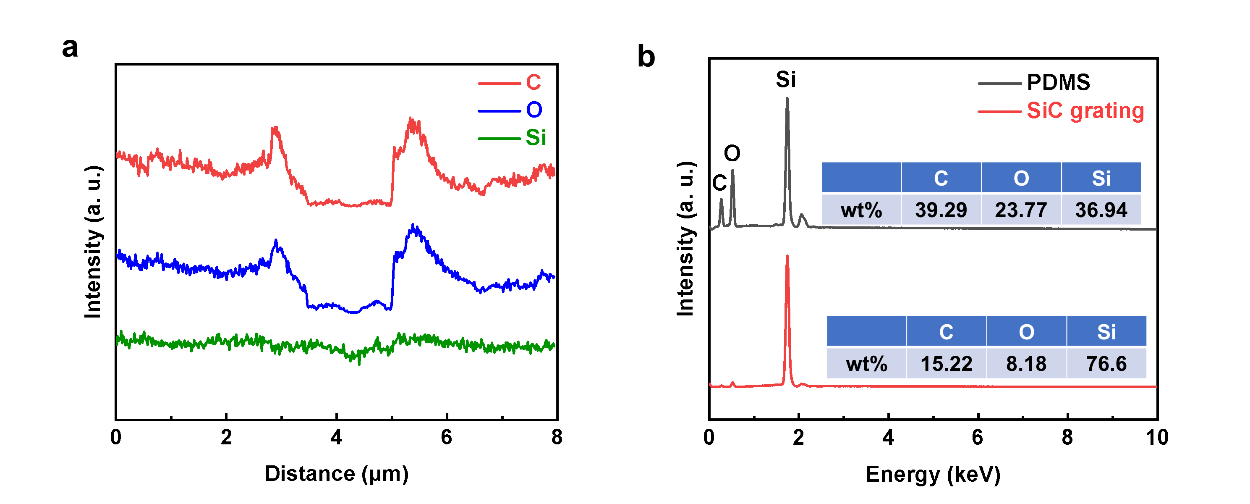


**Fig. S7** (a) EDS line scanning profiles. (b) EDS spectra of the initial PDMS and SiC grating.


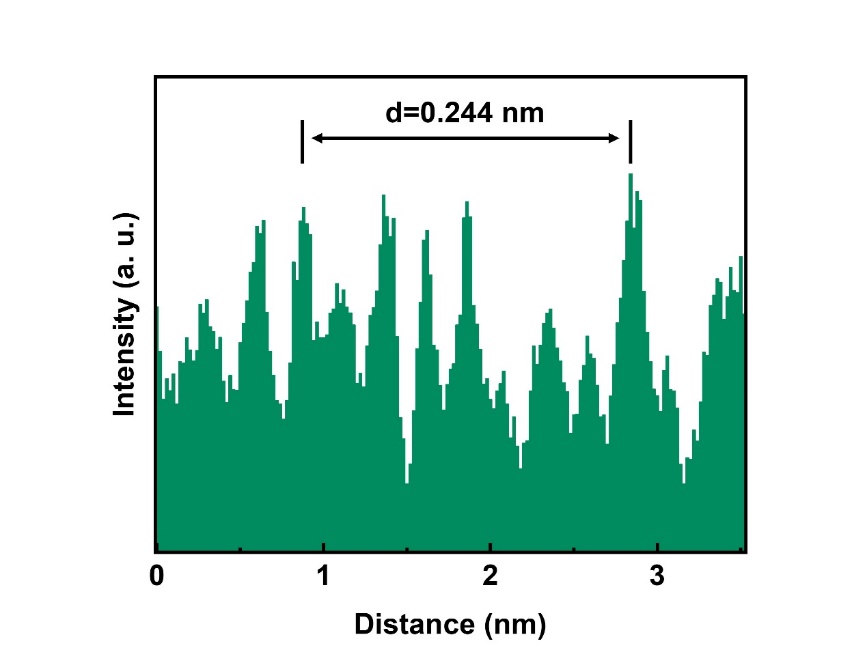


**Fig. S8** Lattice distance calculated from a digital micrograph.


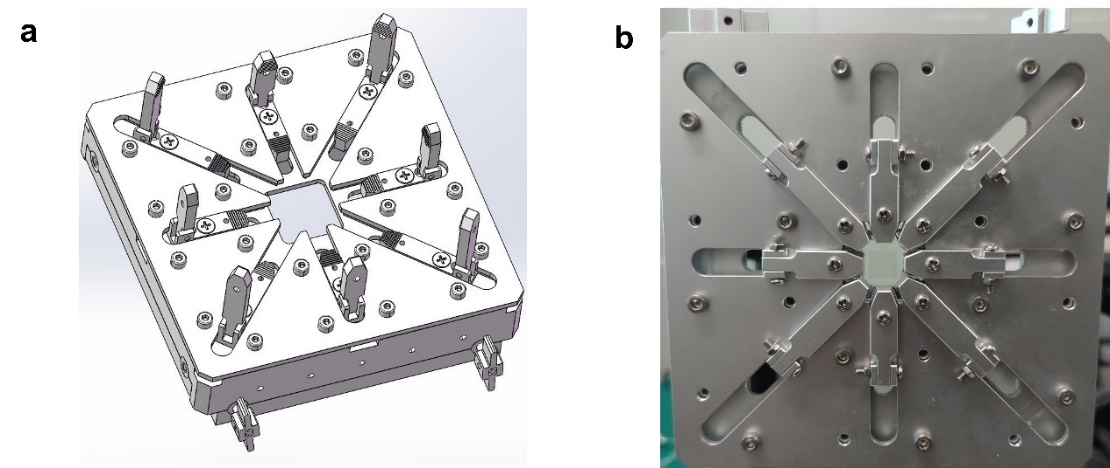


**Fig. S9** (a) Design and (b) photograph of the custom-designed square stretching device.


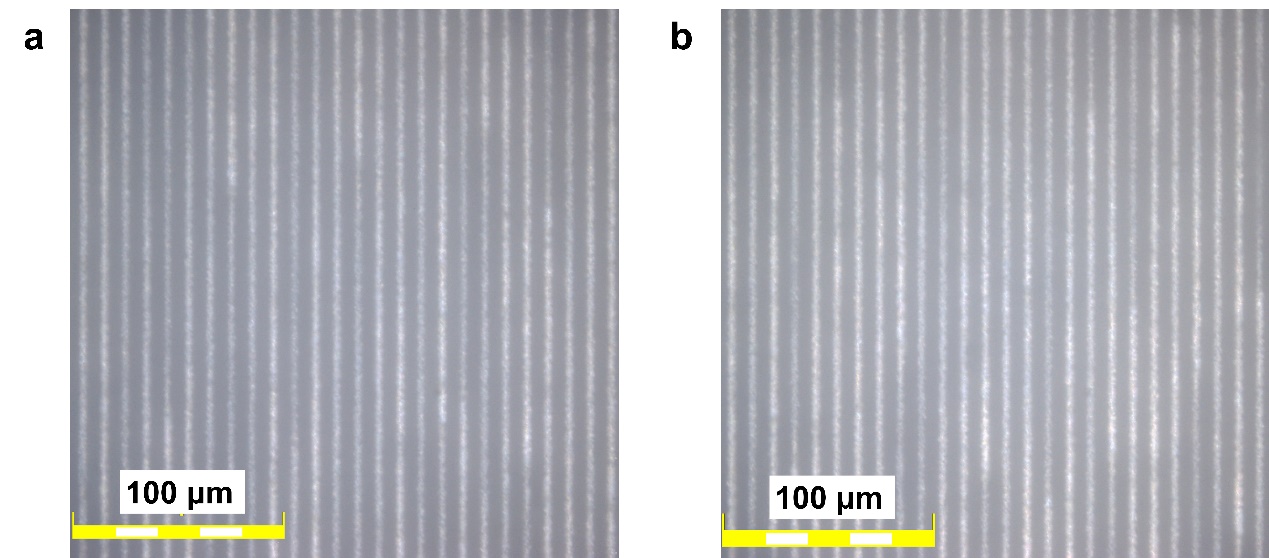


**Fig. S10** CLSM images of embedded SiC gratings (a) before and (b) after 1000 stretching-releasing cycles at 38% strain. No visible cracking or delamination is observed, indicating good mechanical stability of the grating structures during repeated deformation.


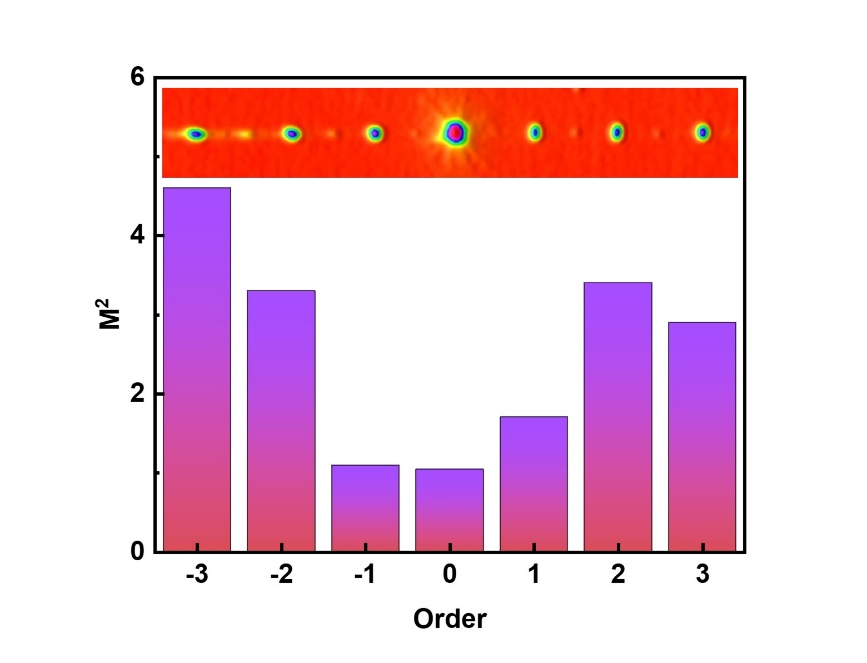


**Fig. S11** Beam quality of the surface 1D diffraction spots.


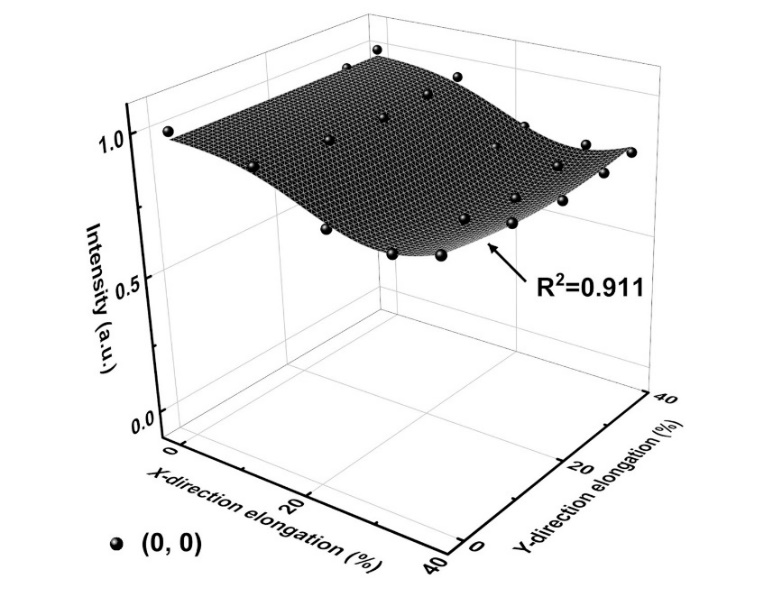


**Fig. S12** Dependence of the (0^th^, 0^th^)-order 2D diffraction efficiency on the mechanical elongation.


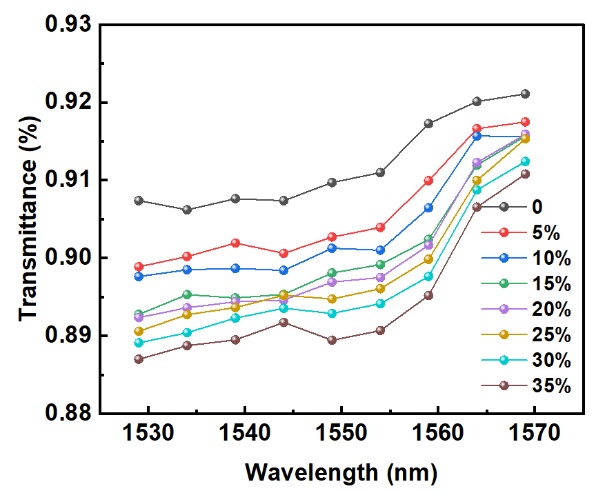


**Fig. S13** SiC grating's transmittance from 1530-1550 nm during dynamic tuning


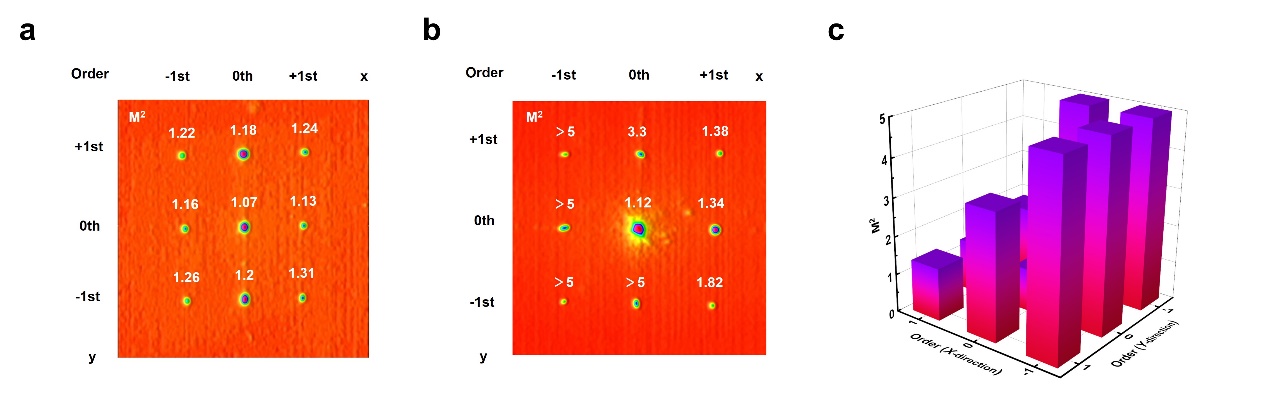


**Fig. S14** (a) Light field distributions of diffraction spots obtained using an internal SiC grating. (b) Light field distributions and (c) 2D beam quality of diffraction spots obtained using surface SiC gratings.


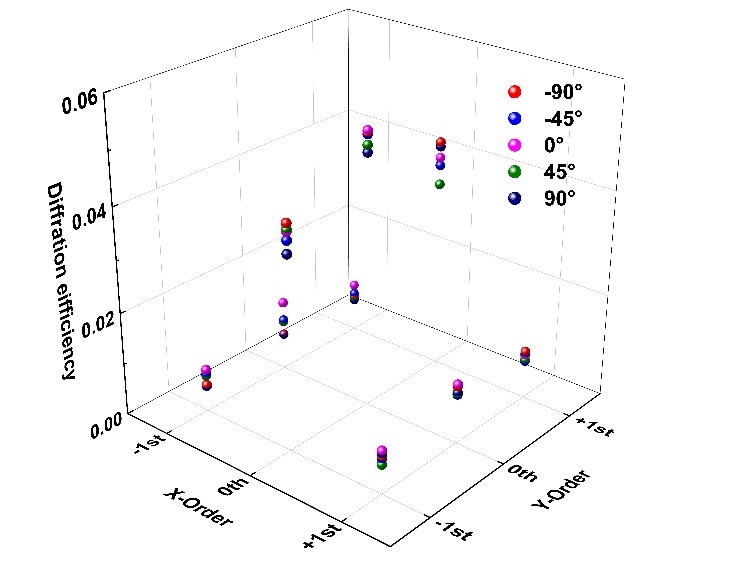


**Fig. S15** Diffraction efficiency of 2D scanning system under different polarization states of laser sources


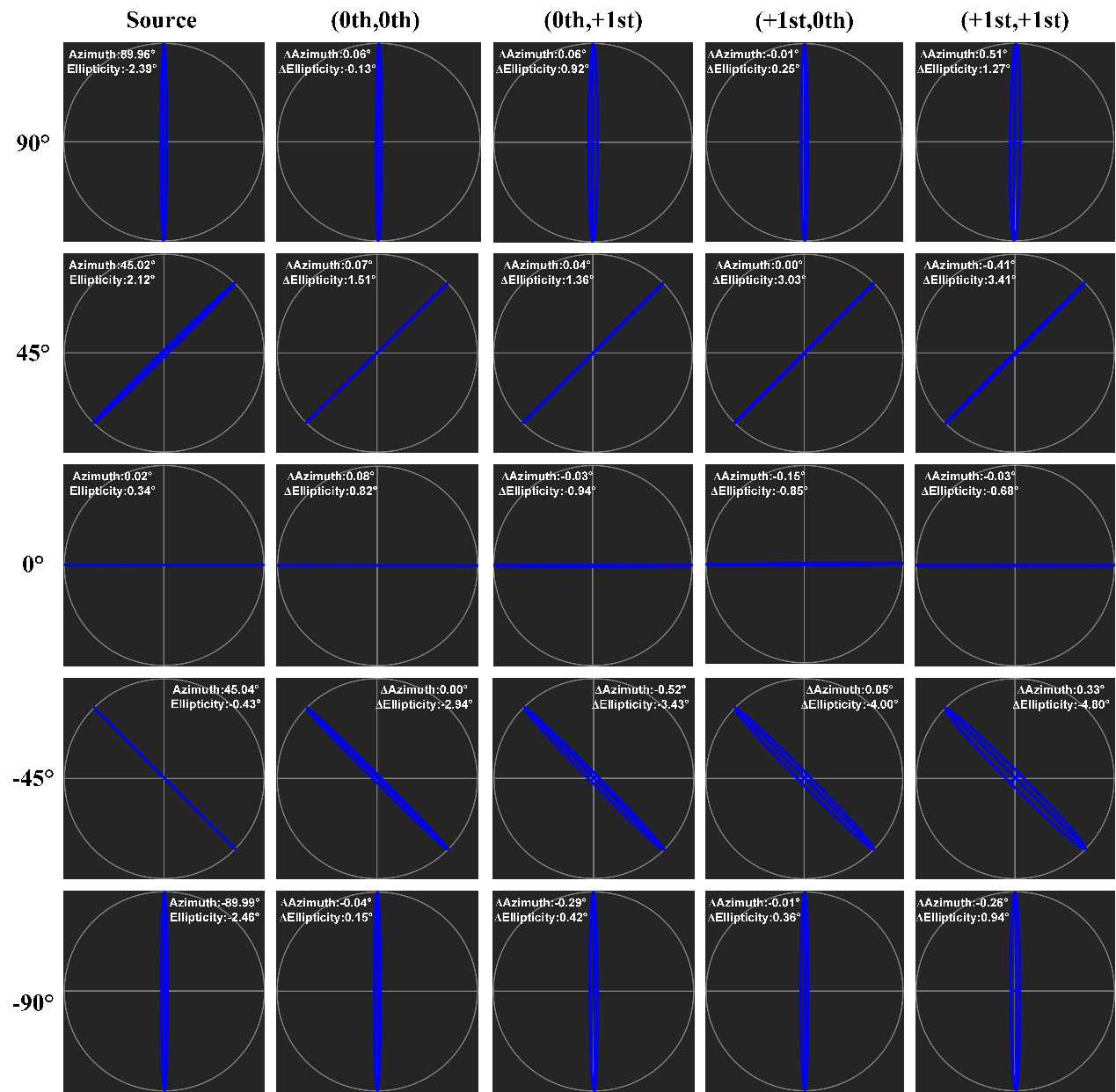


**Fig. S16** Polarization evolution of 2D scanning system under different polarization states of laser sources


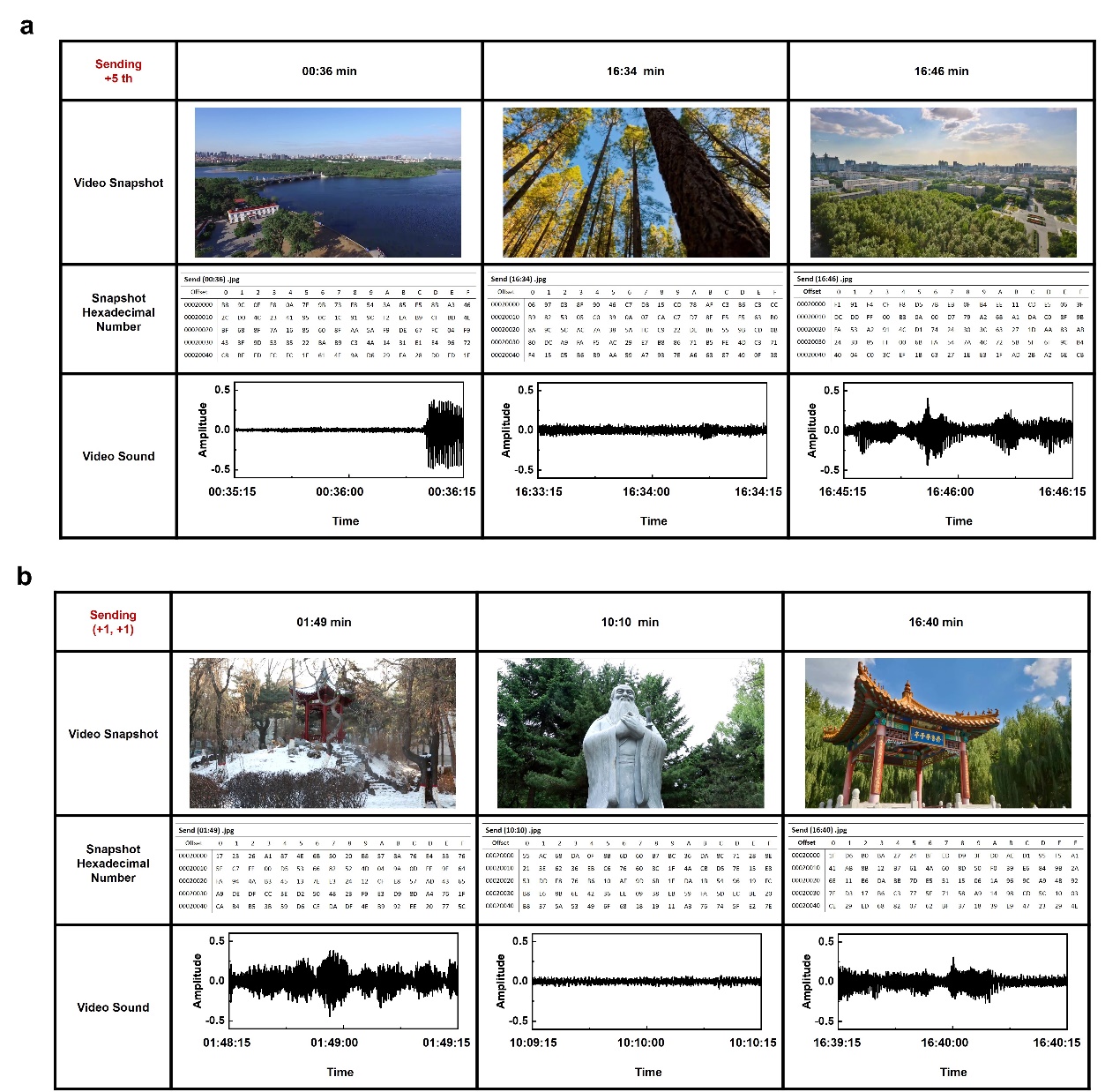


**Fig. S17** Sending data of video snapshots, hexadecimal number snapshots and video sound. (a) Space laser communication system based on a single grating (b) Space laser communication system based on cascaded gratings.


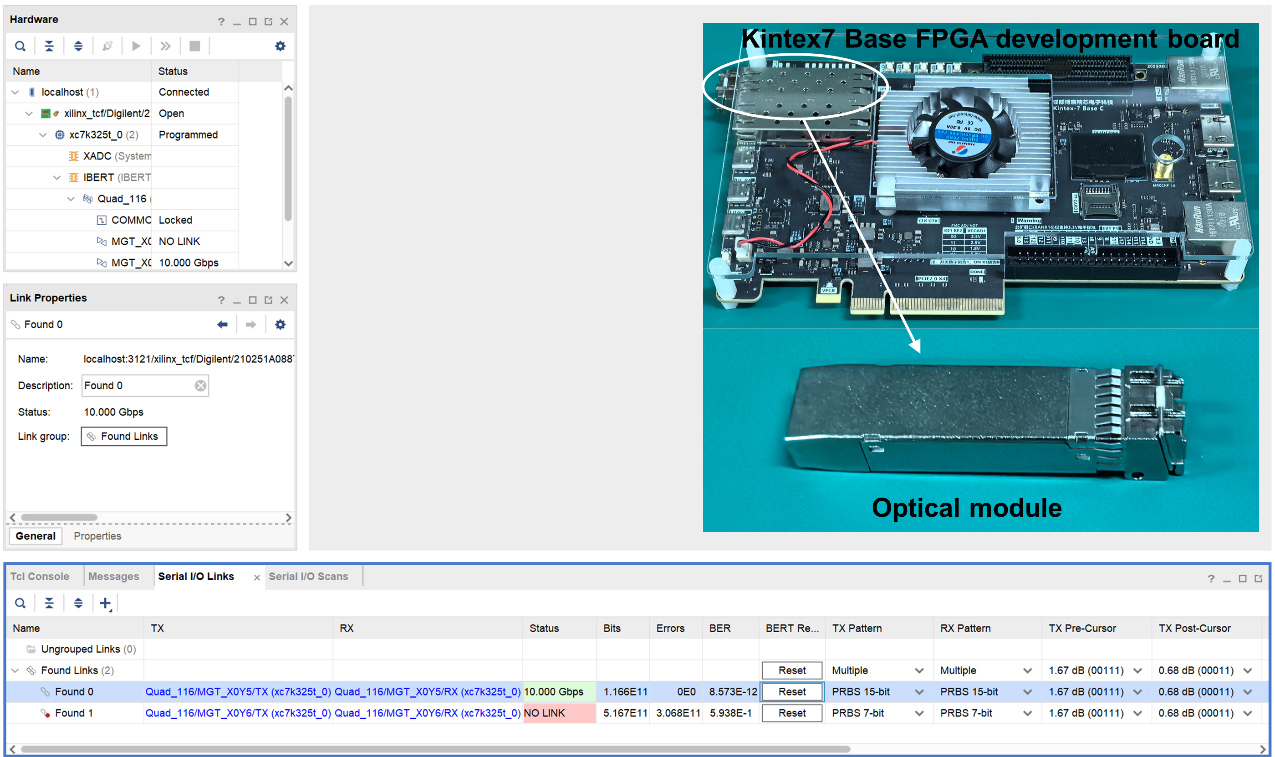


**Fig. S18** Physical object and software interface of the FPGA development board.


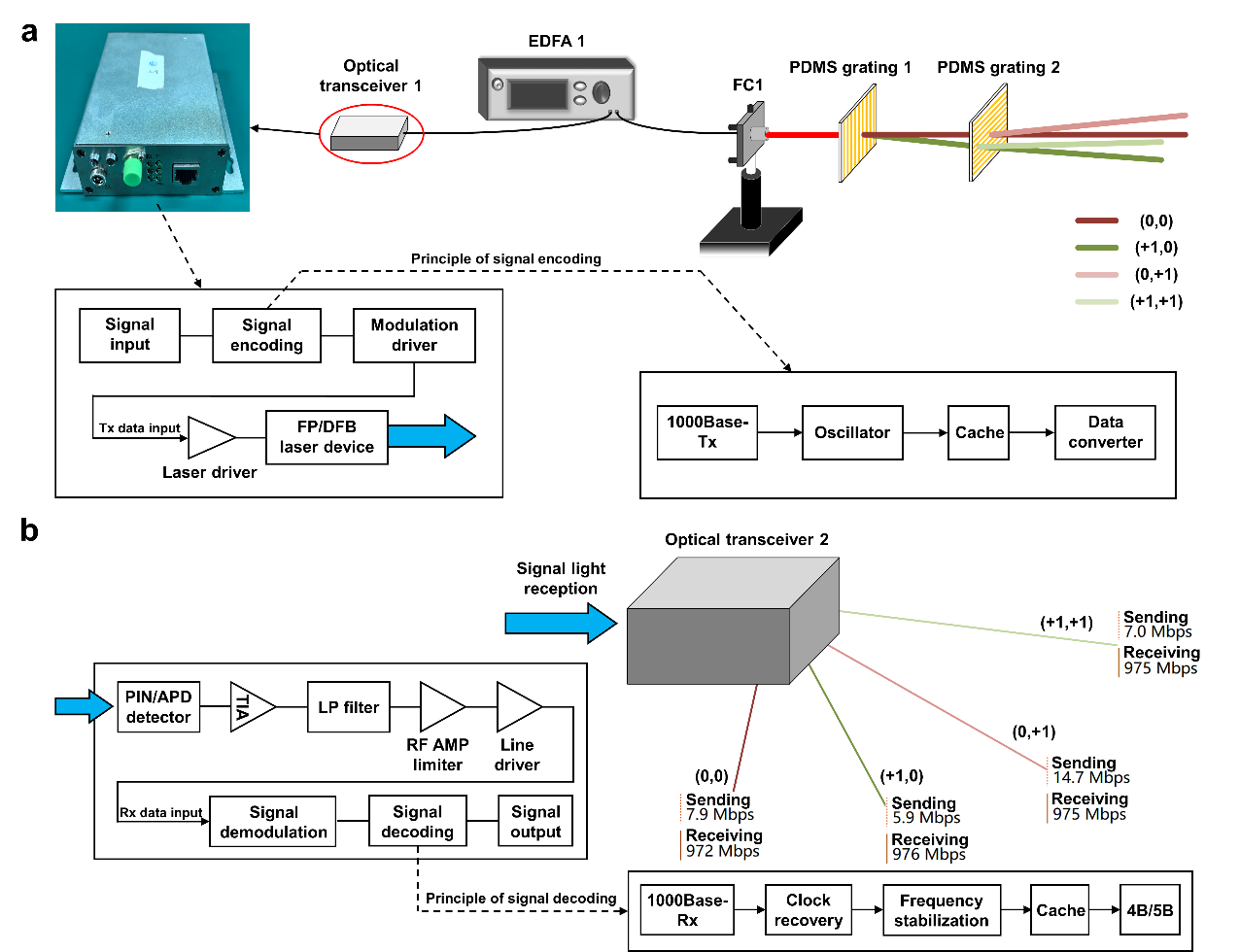


**Fig. S19** Schematic diagram of video transmission using an optical transceiver. (a) Transmitting end. (b) Receiving end.

**
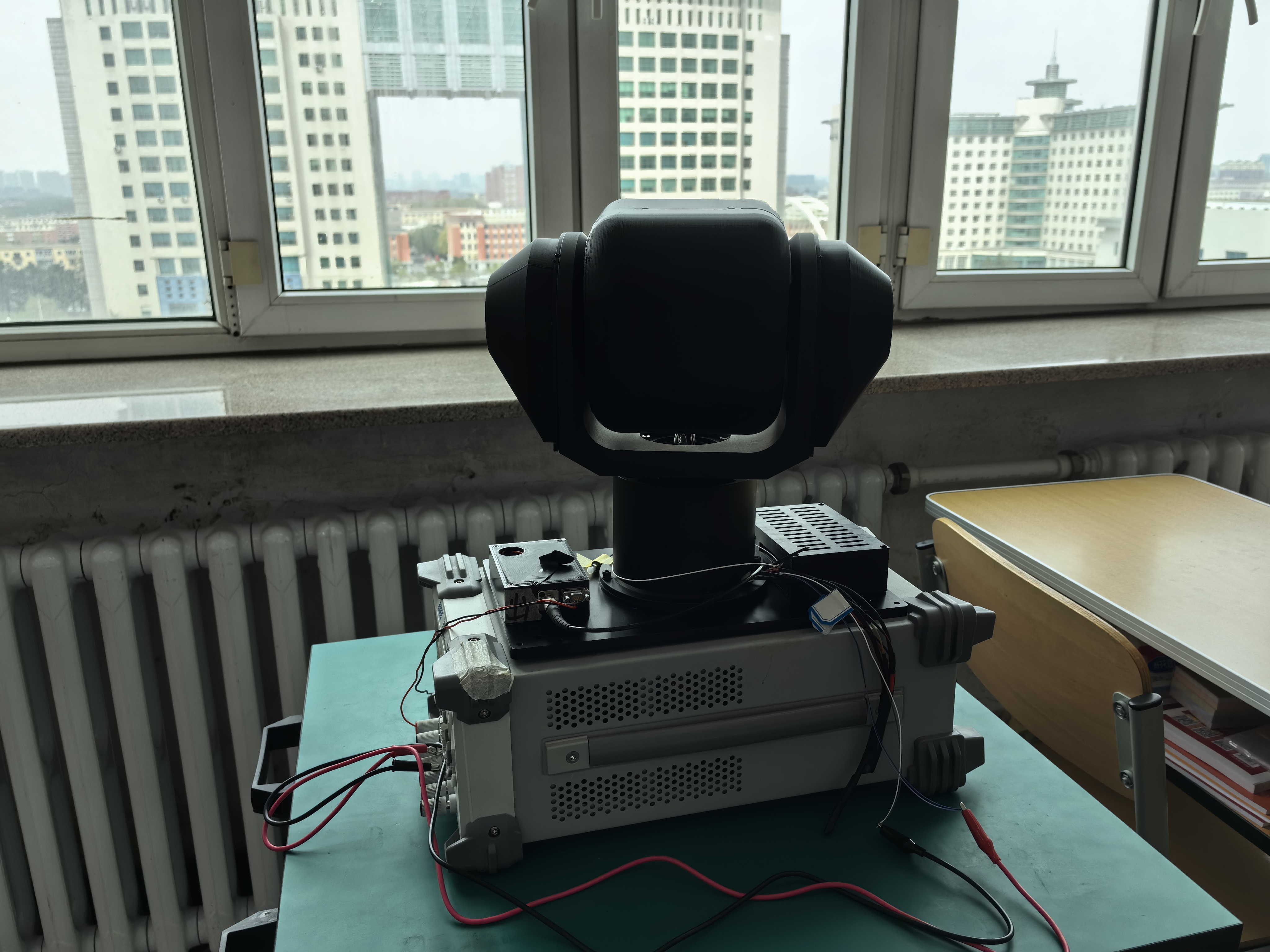
**

**Fig. S20** Home-built FSOC terminal for small platforms.


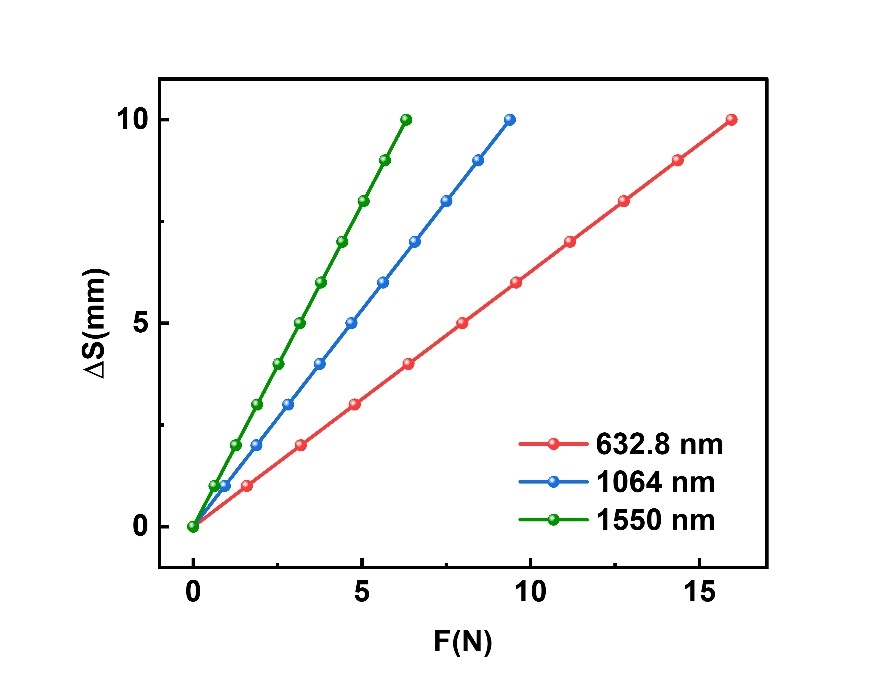


**Fig. S21** Relationship between the variation in the diffraction spot and tensile force.


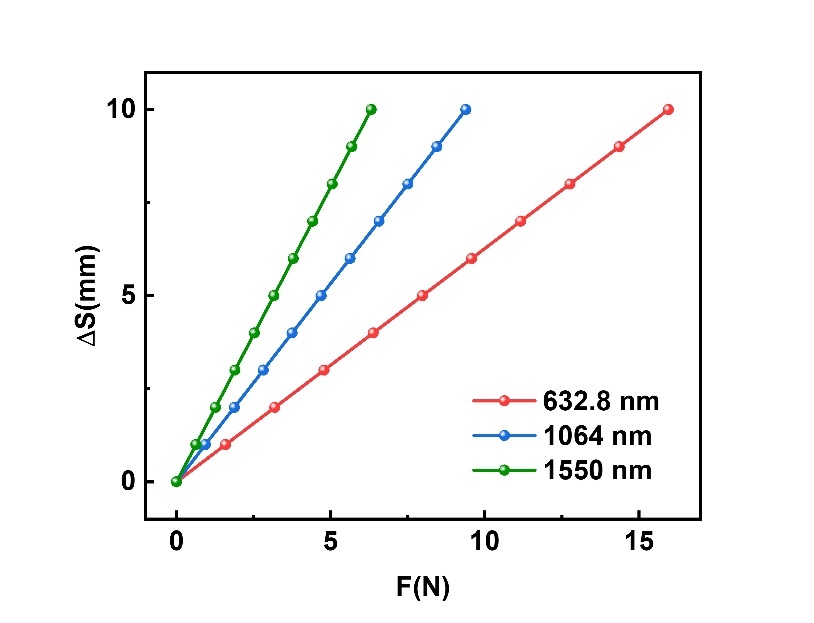


**Fig. S22** Relationship between the variation in the diffraction spot position and stretching force.

**
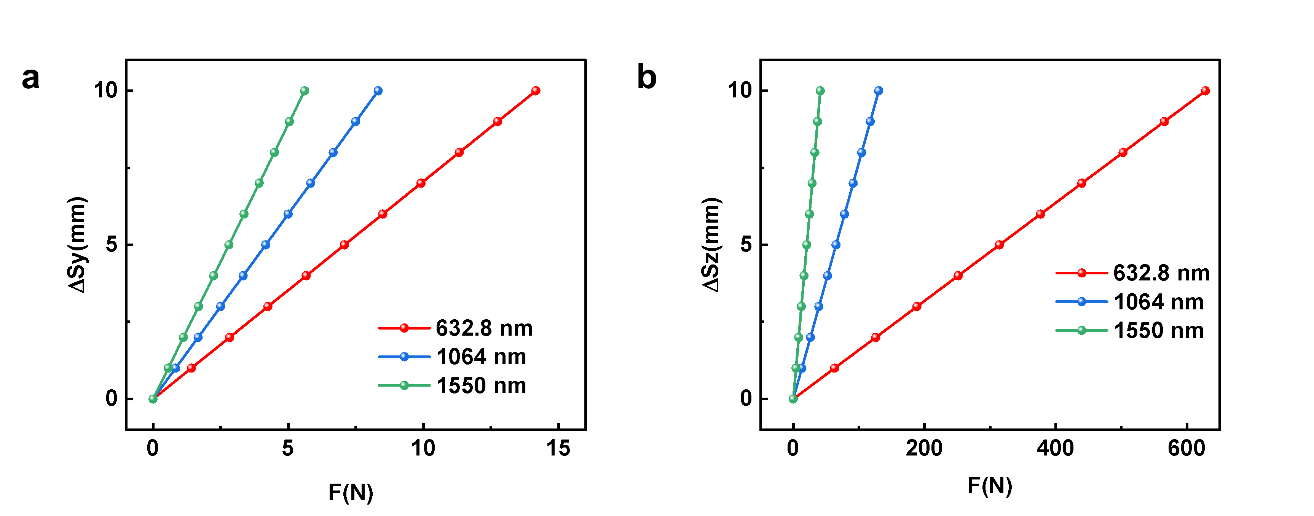
**

**Fig. S23** Relationship between the stretching force and the (a) change in the spot position ΔSy and (b) change in the spot position ΔSz.

**Table 1.** Comparison of point-to-multipoint FSOC performances with other micro-optoelectronic devices.

| Structures | Established nodes | Scenarios | Distance (m) | References |
| --- | --- | --- | --- | --- |
| Double prism | 1-to-3 | Laboratory | 0.76 | 12 |
| Diffuse surface | 1-to-3 | Laboratory | 1.5 | 22 |
| Metasurface | 1-to-1 | Laboratory | 1.2 | 19 |
| Metasurface &  liquid crystal on silicon | 1-to-9 | Laboratory | 1.2 | 45 |
| Metasurface | 1-to-9 | Laboratory | 2 | 46 |
| Metalens | 1-to-1 | Laboratory | 0.05 | 47 |
| Metasurface | 1-to-2 | Laboratory | 1.2 | 20 |
|  | 1-to-5 | Indoor | - |  |
| Reconfigurable internal SiC grating | 1-to-9 | Indoor& Outdoor | 225 | This work |

**Table 2.** Link penalty.

| Penalty type | Parameter | Benchmark fixed lens | This work |
| --- | --- | --- | --- |
| Transmitter penalty | $\eta_{ot}$ | Transmitter optical assembly efficiency — a constant, system-dependent parameter. | Exhibiting a fixed functional relationship with the grating stretching ratio (See Response to Comment 5.a) |
| Spatial penalty | $\left[\frac{D}{\theta_{div}\cdot L}\right]^{2}$ | Dependent on the aperture diameter of the receiver unit, transmit beam divergence angle, and link distance. Where the receiver aperture diameter relates to link distance and specific operating conditions.  While the beam divergence angle is a system-fixed specification. | The aperture diameter of the receiver unit and link distance are identical to those in conventional benchmark optical lens systems, while the beam divergence angle $\theta_{div}$is determined by the diffraction order and grating elongation. $\theta_{div}$  within 0-38% elongation:   1. (0 th, 0 th)   290-299 μrad   1. (+1st,0th)   307-316 μrad   1. (0 th,+1 st)   310-330 μrad   1. (+1 st,+1 st)   335-347 μrad |
| Atmospheric turbulence penalty | $\sigma^{2}=1.23C_{n}^{2}\left(\frac{2\pi}{\lambda}\right)^{7/6}L^{11/6}$ | Dependent on the atmospheric refractive index structure constant $C_{n}^{2}$, laser wavelength λ, and link distance L. Independent of the antenna architecture scheme. | Equivalent to benchmark fixed lens |

**Theory for point-to-multipoint free-space optical communication**

**a. Derivation of the spot displacement ΔS as a function of the tensile force under the 1D steering condition**

When a light beam is perpendicularly incident on an internal SiC grating, the grating equation simplifies to:

$d\sin\theta=m\lambda$ (2)

where *d* is the grating line spacing, *θ* is the diffraction angle, *m* is the diffraction order, and *𝜆* is the laser wavelength. For a diffraction order of m=1, the geometric relationship of the diffraction spot position is given by:

$S=L\tan\theta$ (3)

where *L* is the distance from the grating to the light screen, and *S* is the position of the +1^st^ order diffraction spot on the light screen. Based on Hooke's law, the following relationship can be derived:

$E=\sigma/\varepsilon$ (4)

where *E* is the elastic modulus, *σ* is the stress of PDMS, and *ε* is the strain of PDMS. *σ* and *ε* can be calculated as follows:

$\sigma=F/A$ (5)

$\varepsilon=\Delta d/d$ (6)

where, *A* is the cross-sectional area of the internal SiC grating, *F* is the force applied to the grating, *Δd* is the variation in the grating period, and *d* is the period of the internal SiC gratings. The strain of the grating can be approximated as the strain of each grating line, as the surface of the internal SiC grating exhibits a uniform reticle structure. Consequently, Eq. 6 can be derived from Eqs. 4-6

$\Delta d/d=F/EA$ (7)

Next, derivatives are taken on both sides of Eq. 2 and Eq. 3:

$\Delta\theta\cdot\cos(\theta)=-\frac{\lambda}{d^{2}}\cdot\Delta d$ (8)

$\Delta s=\frac{L}{\cos^{2}\theta}\Delta\theta$ (9)

where *Δθ* is the variation in the diffraction angle, and *Δs* is the change in the spot position. Finally, the relationship between the force *F* and the change in the spot position is derived from Eqs. 7-9

$F=-EA\frac{d\cos^{3}\theta}{\lambda L}\Delta s$ (10)

Fig. S21 shows the relationship between the applied force *F* and the change in spot position *Δs* for light source wavelengths of 632.8 nm, 1064 nm and 1550 nm. When the mixing ratio of the PDMS solution is 10:1, the elastic modulus *E* is 7.5 × 10^5^ Pa. The distance between the screen and the grating is 15.8 cm, and the diffraction angles *θ* are 3.727° at 650 nm, 6.108° at 1064 nm and 8.917° at 1550 nm. The grating period *d* is 10 µm, and the cross-sectional area *A* is 21.4 mm^2^. During the stretching process, the change in the diffraction spot position is linear, with the diffraction spot gradually moving closer to the zero-order position. According to Eq. 10, the elastic modulus, spot position, grating size, screen distance, and laser wavelength all influence the scanning results. Additionally, a smaller tensile force is required at 1550 nm than at 632.8 nm and 1064 nm for the diffraction spot to travel the same distance.

**b. Derivation of the spot displacement ΔS as a function of the tensile force under 2D steering conditions**

Since the grating lines of Gratings 1 and 2 are spatially perpendicular to each other, the ±1^st^-order diffracted beams generated by Grating 1 pass through Grating 2, whereas the 0^th^-order diffracted beam continues to propagate along the original path. Consequently, the diffraction spots are distributed along the X-axis. The grating equation for Grating 2 is as follows:

 (11)

where *d_2_* is the period of Grating 2 and *θ_z_* is the diffraction angle of the beam passing through Grating 2. When *m_1_*=*m_2_*=1, the distance Sy between (0^th^, 0^th^) and (+1^st^, 0^th^) and the distance *S_z_* between (+1^st^, 0^th^) and (+1^st^, +1^st^) are as follows:

 (12)

 (13)

The theoretical analysis of the steering behaviour of cascaded gratings can be divided into the following two cases:

First, when only Grating 2 is stretched, the period *d_1_* of Grating 1, the diffraction angle *θ_y_* and the distance *S_y_* between the (0^th^, 0^th^)- and (+1^st^, 0^th^)-order diffraction spots remain constant. Taking the derivatives of both sides of Eq. 11 and Eq. 13:

 (14)

 (15)

Finally, the relationship between the force *F* and the change in the spot position *ΔS_z_* is derived via Eqs. 7, 14 and 15:

 (16)

The relationship between the stretching force *F* and the variation in the diffraction spot position *ΔS_z_* for light source wavelengths of 632.8 nm, 1064 nm, and 1550 nm is shown in Fig. S22. The elastic modulus *E* used in the simulation is consistent with that used in the 1D scanning simulation. The grating periods *d_1_* and *d_2_* are both 10 µm. According to the grating equation, the diffraction angles *θ_y_* and *θ_z_* are 3.727° at 650 nm, 6.108° at 1064 nm, and 8.917° at 1550 nm. The distance between Gratings 1 and 2 (*L_1_*) is 2 cm, and the distance between the screen and Grating 2 (L_2_) is 1 m. As shown in Fig. 4, the diffraction spot position linearly changes during stretching, gradually moving closer to the Y-axis. Additionally, less force is required at 1550 nm than at 650 nm and 1064 nm for the diffraction spot to travel the same distance.

Second, when only Grating 1 is stretched, the grating period *d_2_* and diffraction angle *θ_z_* remain constant. The distances *S_y_* and *S_z_* between the (+1^st^, 0^th^)-and (+1^st^, +1^st^)-order diffraction spots both change. For *S_y_*, derivatives are taken on both sides of Eq. 2 and Eq. 12:

 (17)

 (18)

The relationship between the force *F* and the change in spot position *ΔS_y_* is derived via Eqs. 7, 17, and 18:

 (19)

For *S_z_*, derivatives are taken on both sides of Eq. 13, with the right-hand side differentiated with respect to *θ_y_*:

 (20)

The relationship between the force *F* and the change in spot position *ΔS_z_* is derived via Eqs. 7, 19, and 20:

 (21)

Figs. S23 shows the relationships between the force *F* and the changes in the spot positions *ΔS_y_* and *ΔS_z_*, respectively. As shown in Fig. 3i, the diffraction spot linearly changes during stretching, gradually approaching the X- and Y-axes. However, the change in *ΔS_z_* is relatively small, and if the distance *L_2_* between Grating 2 and the screen is sufficiently small, the movement in the Y-axis direction becomes negligible for point scanning applications. Additionally, less force is required at a wavelength of 1550 nm than at 632.8 nm and 1064 nm to achieve the same spot displacement. Thus, 1550 nm light sources are more suitable for these experiments.
